# High-dose multi-strain *Bacillus* probiotics enhance treatment and reduce antibiotic usage in children with persistent diarrhea through immune and microbiota modulation

**DOI:** 10.1038/s41598-025-15199-y

**Published:** 2025-08-18

**Authors:** Ha Thuy Dang, Thuy Thi Bich Phung, Dien Minh Tran, Anh Thi Phuong Bui, Yen Hai Vu, Minh Thi Luong, Hang Minh Nguyen, Huong Thi Trinh, Hao Thi Ngoc Vo, Trang Thi Thu Nguyen, Anh Hoa Nguyen, Pham Dinh Tung, Linh Huyen Tran, Anh Thi Van Nguyen

**Affiliations:** 1 Department of Gastroenterology, Vietnam National Children’s Hospital, No. 18/879 Lathanh, Lang, Hanoi, Vietnam; 2 Department of Surgical Intensive Care Unit, Vietnam National Children’s Hospital, No. 18/879 Lathanh, Lang, Hanoi, Vietnam; 3 Department of Molecular Biology for Infectious Diseases, Vietnam National Children’s Hospital, No. 18/879 Lathanh, Lang, Hanoi, Vietnam; 4 Spobiotic Research Center, ANABIO R&D Ltd. Company, No. 22, Lot 7,8 Van Khe Urban, Ha Dong, Hanoi, Vietnam; 5 LiveSpo Pharma Ltd. Company, N03T5, Ngoai Giao Doan Urban, Xuan Dinh, Hanoi, Vietnam; 6 Faculty of Mathematics-Mechanics-Informatics, VNU University of Science, Vietnam National University, 334 Nguyen Trai, Thanh Xuan, Hanoi, Vietnam; 7 Institute of Biology, Vietnam Academy of Science and Technology, 18 Hoang Quoc Viet, Nghia Do, Hanoi, Vietnam

**Keywords:** Persistent diarrhea, Probiotic, *Bacillus*, Spore, Antibiotic, Cytokine, Microbiota, Randomized controlled trials, Diarrhoea, Paediatric research, Clinical microbiology, Microbiome, Cytokines

## Abstract

Persistent diarrhea remains medical challenge to date regarding both its etiology and therapeutic interventions. Here, we conducted a randomized, double-blind, controlled clinical trial to evaluate the effectiveness of high-dose multi-strain *Bacillus* spore probiotics (LiveSpo DIA30) containing *B. subtilis*,* B. clausii*, and *B. coagulans* at 5 billion CFU/5 mL ampoule, in the supportive treatment of persistent diarrhea in children. Our findings revealed a significant 3-day shorter recovery period, 1.60-fold enhanced efficacy, and a 9.47-fold increase in odds (all *p-*values < 0.0001) for effectively resolving diarrhea by day 5 with the *Bacillus* spores. Notably, the group receiving LiveSpo DIA30 (Dia30 group) experienced a reduction in antibiotic treatment duration by 2 days compared to the Control group, equivalent to a 25% decrease in antibiotic usage. After 5 days of treatment, LiveSpo DIA30 demonstrated significant reductions in elevated blood pro-inflammatory cytokines, including IL-17 (26.62%; *p* = 0.0178), IL-23 (25.13%; *p* = 0.0256), TNF-α (19.09%; *p* = 0.038), and in fecal sIgA (24.24%; *p* = 0.0433). Analysis of 16S rRNA metagenome revealed that Dia30 group exhibited a notable increase in density of Actinomycetota and Bacillota phylum, along with Actinomycetaceae, Lactobacillaceae, and Streptococaceae families. *Lacticaseibacillus rhamnosus*, a beneficial gut species, was not detectable at day 0 but reached a density of 0.91% (*p* = 0.015) in Dia30 group by day 5. Additionally, Dia30 group showed a significant reduction in density of the Proteobacteria phylum, Enterobacteriaceae family, and harmful species *Escherichia fergusoni* (682.8-fold; *p* = 0.011). In conclusion, this clinical trial presents robust clinical evidence, supported by laboratory testing data, demonstrating the efficacy of multi-strain and high-concentration *Bacillus* spore probiotics in rapidly alleviating symptoms and reducing antibiotic usage in children with persistent diarrhea. This is archived by improving the native gut microbiota and modulating immunological responses.

**Trial registration**: ClinicalTrials.gov, Identifier No: NCT05812820, 14/4/2023.

**Supplementary Information:**

The online version contains supplementary material available at 10.1038/s41598-025-15199-y.

## Introduction

The World Health Organization (WHO) defines persistent diarrhea as lasting between 2 and 4 weeks and excludes cases of chronic diarrhea caused by conditions like celiac disease, food-related enteropathies, and congenital enteropathies. Persistent diarrhea, constituting around 3–20% of diarrhea cases in children under the age of five, can lead to severe dehydration, malnutrition, and hospitalization if not addressed promptly^1–3^. In tropical countries, approximately 5–10% of acute diarrhea episodes progress to persistent diarrhea, responsible for half of all diarrhea-related deaths,^2,4^. During COVID-19 outbreaks, increased diarrhea rates were observed in children, often linked to higher systemic inflammation. This condition leads to malnutrition, creating a vicious cycle impacting a child’s growth^5^. To date, most researchers consider persistent diarrhea as an infectious disease due to the invasion of pathogens into the intestinal tract, including bacteria, viruses, or/and disease-causing parasites^6^. Additionally, diarrhea can also be characterized as one of the adverse effects of antibiotics, commonly referred to as Antibiotic-Associated Diarrhea (AAD). Antibiotics deplete beneficial bacteria, causing overgrowth of pathogenic bacteria, resulting in persistent diarrhea and poor nutrient absorption. Cephalosporins, penicillin, and clindamycin, administered either orally or intravenously, carry a substantial risk of inducing AAD^7^. It is due to their impacts on the mucosal layer, tight junctions between intestinal epithelial cells and bacterial protease activity, all contributing to disrupted functioning of the gut mucosal barrier^8^. For cases with unidentified etiology, there is evidence to suggest that persistent diarrhea is linked to both intestinal immune responses and dysbiosis within the gut^2,3,9^. Diarrhea, although not always linked to acute inflammation, is strongly associated with intestinal inflammation, characterized with increased pro-inflammatory cytokines (IL-1β, IL-6, IL-8, IFN-γ, TNF-α) and reduced anti-inflammatory cytokines (IL-4, IL-10)^9–11^together with infiltration of Th17 and IgA-producing B lymphocytes in the gut^12–15^. The gut microbiota, the largest ecosystem in the human body, consists of a complex and dynamic community of microorganisms, and its balance is critical to maintaining a healthy digestive system. During the “first 1000 days of life”, a child’s gut microbiota begins to develop with high individualization and low diversity. Therefore, any disruption during this period leads to an imbalance in the gut microbiota. There is increasing evidence that changes in the composition and function of the gut microbiota are early events in a range of childhood diseases, including persistent diarrhea^16–18^.

To treat enteric pathogens in persistent diarrhea, the World Health Organization (WHO) recommends combined oral and intravenous antibiotic therapy when single antibiotics fail. However, prolonged high-dose antibiotics can cause dysbiosis and antibiotic-resistant bacteria, leading to malabsorption and mucosal barrier dysfunction. Consequently, persistent diarrhea is challenging to resolve^6,15^. In addition to antibiotic therapy, probiotics are frequently used to promote gut health by modulating metabolic processes, immune function, and the equilibrium of gut microbiota^16^. Numerous clinical trials have explored the efficacy of *Lactobacillus* and *Bifidobacterium* probiotic species in promoting gastrointestinal health and reducing the severity and frequency of diarrhea caused by various intestinal pathogens, such as *Clostridium difficile*, different strains of *E. coli*, and rotavirus^18–20^. In compared to these probiotics, *Bacillus* probiotics have an advantage in forming endospores resistant to harsh environmental factors like heat, acidic gastric conditions, and bile. This unique character allows *Bacillus* probiotics to endure the digestive tract’s challenging conditions and arrive intact in the intestines, where they canoffer health benefit. Among safe *Bacillus* species, *B. subtilis*,* B. clausii*, and *B. coagulans* are commercially recommended for restoring disrupted intestinal microbiota in both children and adults. However, probiotic studies thus far have mainly focused on mild gastrointestinal disturbances or acute diarrhea^21–23^but not persistent diarrhea. The doses of probiotics approximately 2–4 billion CFU per day as commonly recommended by manufacturers may not yield significant results for persistent diarrhea. Our recent study demonstrated that the utilization of high-dose *Bacillus clausii* liquid-form probiotics, known for their safety at 8–12 billion CFU per day, effectively reduced typical symptoms of persistent diarrhea by regulating the gut immune system^24^.

In this study, we conducted a randomized, double-blind, and controlled clinical study to assess the efficacy of a liquid-form *Bacillus* spore probiotics, LiveSpo DIA30, containing *B. subtilis* ANA48, *B. clausii* ANA39, and *B. coagulans* ANA40 strains at a concentration of 5 billion CFU/5 mL per ampoule, in children aged 3 to 24 months with persistent diarrhea. We hypothesized that using multi-strains, rather than a single-strain, *Bacillus* probiotics at high concentration can achieve faster and more effective symptom relief. This would help reduce the antibiotic reliance, and expedite the recovery of gut microbiota as well as regulating the gut immune system. The probiotics were administered orally at a high dosage of 20–30 billion CFU per day. This dosage falls within the allowable dose mentioned by the World Gastroenterology Organization (WGO)^25^ and the manufacturer’s instruction usage for severe diarrhea. Throughout the study, we monitored three typical symptoms of diarrhea and tracked the duration of antibiotic usage, aiming to estimate the potential reduction in antibiotic dependence resulting from probiotic supplementation. Additionally, we evaluated the changes in pro-inflammatory and anti-inflammatory cytokines in blood of patients, as well as accessed IgA levels and gut microbiota in stool samples, comparing day 5 or end of treatment with day 0 before treatment initiation.

## Results

### Trial design and patient baseline demographics, clinical and subclinical characteristics

**Fig. 1:**
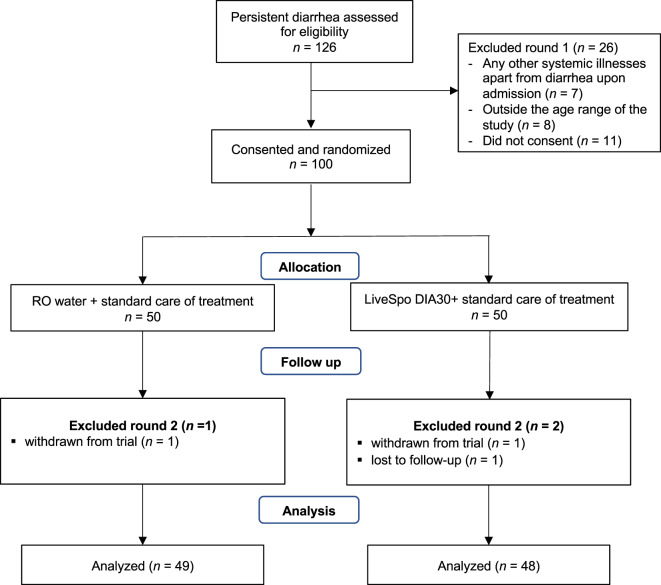
A flowchart illustrating study design including recruitment of participants, assessments for exclusion, treatment and data analysis. The study was carried out from April 15, 2023 to December, 25 2023.

A total of 126 participants underwent eligibility screening from April 15, 2023 to December 25, 2023. Among them, 100 eligible persistent diarrhea patients were randomly divided into two groups: the standard-of-care group (Control group; *n* = 50) and the group receiving LiveSpo DIA30 (Dia30 group; *n* = 50) (Fig. 1–exclusion round 1). During the standard 5-10-day follow-up period post-treatment, one participant from Control and two from Dia30 groups were subsequently excluded, resulting in a final analysis cohort of 49 and 48 and participants in each group, respectively (Fig. 1–exclusion round 2).

The data in Table 1 show that the demographics of children in the two study groups do not differ significantly (all *p* values > 0.05) in terms of age, gender, history of maternal COVID-19 infection, weight-for-age Z-scores, and height-for-age Z-scores. We noted that 28.57% in the Control group and 37.50% in the Dia30 groups had used antibiotics before the onset of diarrhea (*p* = 0.3497). Prior to the onset of diarrhea, the most common antibiotics to treat acute respiratory or intestinal infections were Biseptol, Cefixime, and Augmentin. Before treatment, the baseline clinical parameters, including duration of diarrhea, frequency of daily bowel movements equal or more than 3 times per day, the presence of fecal mucus, and diapered infant stool scale types 4-5B, displayed no significant disparities between the two groups (all *p* values > 0.05). As shown in Table 2, the two groups also exhibited similar frequency of bowel movements across three categorized levels (3–5 stools a day, 6–7 stools a day, and *≥* 8 stools a day) (*p* values > 0.05).

**Table 1:** Demographic characteristics and fecal pathogenic microbial infections in children with persistent diarrhea before treatment.

Characteristic	Control group (*N* = 49)	Dia30 group (*N* = 48)	*p* value
Age (months)	5 ± 2.5	5 ± 3.88	0.4006^c^
Gender			
Male *n* (%)	29 (59.18)	28 (58.33)	0.9322^b^
Female *n* (%)	20 (40.82)	20 (41.67)	
Covid-19 infection			
Mother *n* (%)	22 (44.90)	16 (33.33)	0.2434^b^
Children *n* (%)	4 (8.16)	4 (8.33)	> 0.9999^a^
Weight			
Average ± SD	7.38 ± 1.33	7.47 ± 1.33	0.7583^c^
Weight for age Z-score			
<-2SD *n* (%)	1 (2.04)	1 (2.08)	> 0.9999^a^
≥-2SD and ≤ 2SD *n* (%)	47 (95.92)	46 (95.83)	> 0.9999^a^
> 2SD *n* (%)	1 (2.04)	1 (2.08)	> 0.9999^a^
Height			
Average ± SD	65.70 ± 4.98	67.22 ± 6.58	0.2015^d^
Height for age Z-score			
<-2SD *n* (%)	2 (4.08)	5 (10.42)	0.2682^a^
≥-2SD and ≤ 2SD *n* (%)	45 (91.84)	41 (85.42)	0.356^a^
> 2SD *n* (%)	2 (4.08)	2 (4.17)	> 0.9999^a^
Antibiotic usage before diarrhea onset n (%)	14 (28.57)	18 (37.50)	0.3497^b^
Median days of persistent diarrhea before treatment	20	20	0.1086^c^
24-pathogen intestinal microbial real-time RT-PCR test n (%)			
Patients with bacterial infections	12 (24.49)	10 (20.83)	0.6672^b^
*Campylobacter* spp.	2 (4.08)	2 (4.17)	> 0.9999^a^
*Clostridium difficile (toxin A/B)*	4 (8.16)	4 (8.33)	> 0.9999^a^
*Enteroaggregative E. coli*	3 (6.12)	2 (4.17)	> 0.9999^a^
*Enteropathogenic E. coli*	2 (4.08)	1 (2.08)	> 0.9999^a^
*Enterotoxigenic E. coli*	1 (2.04)	0 (0.0)	> 0.9999^a^
*Plesiomonas shigelloides*	1 (2.04)	2 (4.17)	0.6171^a^
*Salmonella* spp.	3 (6.12)	2 (4.17)	> 0.9999^a^
Patients with viral infections	3 (6.12)	4 (8.33)	0.7147^a^
Astrovirus	0 (0.00)	1 (2.08)	0.4948^a^
Norovirus GI	0 (0.00)	1 (2.08)	0.4948^a^
Norovirus GII	3 (6.12)	2 (4.17)	> 0.9999^a^
Total number of infected patients	14 (28.57)	13 (27.08)	0.8701^b^

^a^Fisher’s Exact test; ^b^Chi-Square test; ^c^ Mann-Whitney test; ^d^ *t-*test.

**Table 2:** Clinical and fecal subclinical characteristics in persistent diarrhea children before, during, and after treatment.

Characteristic	Control group (*N* = 49)			Dia30 group (*N* = 48)			*p* value		
	Day 0	Day 3	Day 5	Day 0	Day 3	Day 5	Day 0	Day 3	Day 5
Typical symptoms									
Stools a day of ≥ 3 *n*(%)	49 (100)	41 (83.67)	38 (77.55)	48 (100)	34 (70.83)	14 (29.17)	> 0.9999^a^	0.1311^b^	**< 0.0001** ^**b**^
3–5 stools a day	25 (51.02)	30 (61.22)	33 (67.35)	24 (50.0)	32 (66.67)	13 (27.08)	0.9199^b^	0.5768^b^	**< 0.0001** ^**b**^
6–7 stools a day	18 (36.73)	10 (20.41)	4 (8.16)	17 (35.42)	1 (2.08)	0 (0.0)	0.8925^b^	**0.0077** ^**a**^	0.1173^a^
≥ 8 stools a day	6 (12.24)	1 (2.04)	1 (2.04)	7 (14.58)	1 (2.08)	1 (2.08)	0.7354^b^	> 0.9999^a^	> 0.9999^a^
Presence of fecal mucus *n* (%)	48 (97.96)	44 (89.80)	34 (69.39)	46 (95.83)	33 (68.75)	12 (25.0)	0.6171^a^	**0.0104** ^**b**^	**< 0.0001** ^**b**^
Diaper stool types 4-5B *n* (%)	47 (95.92)	42 (85.71)	24 (48.98)	44 (91.67)	20 (41.67)	10 (20.83)	0.4357^a^	**< 0.0001** ^**b**^	**0.0037** ^**b**^
Subclinical test for stool n (%)									
Erythrocyte positive (mild to severe)	30 (61.22)	15 (30.61)	5 (10.20)	21 (43.75)	10 (20.83)	1 (2.08)	0.0848^b^	0.2709^b^	0.2041^a^
Leukocyte positive (mild to severe)	44 (89.80)	25 (51.02)	6 (12.24)	46 (95.83)	10 (20.83)	0 (0.0)	0.4357^a^	**0.002** ^**b**^	**0.0266** ^**a**^
pH ≤ 5.5	18 (36.73)	13 (26.53)	10 (20.41)	12 (25.0)	10 (20.83)	6 (12.5)	0.2112^b^	0.5095^b^	0.2941^b^
pH > 5.5	31 (63.27)	36 (73.47)	25 (51.02)	36 (75.0)	38 (79.17)	12 (25.0)			**0.0083** ^**b**^

^a^Fisher’s Exact test; ^b^Chi-Square test.

Regarding the subclinical indicators before treatment, as shown in Table 1, 14 children in the Control (28.57%) and 13 children in Dia30 (27.08%) groups were found to have infections with one to four enteropathogenic microorganisms (*p* = 0.8701). 10 out of 24 screened pathogenic microorganisms responsible for diarrhea were detected in stool samples. Notably, the most common pathogenic microorganisms in both groups were *C. difficile* toxin A/B (8.16–8.33%), *Enteroaggregative E. coli* (4.17–6.12%), *Salmonella* sp. (4.17–6.12%), and Norovirus GII (4.17–6.12%). The results suggest that a large portion of cases (69–74%) presenting with persistent diarrhea may have other underlying causes. Furthermore, there were no significant difference in baseline subclinical characteristics, encompassing the presence of leukocytes and erythrocytes in stool microscopy at day 0, between the two groups (all *p* values > 0.05). Similarly, there were no noteworthy differences in abnormal stool pH values falling below pH 5.5 (*p* = 0.2112) (Table 2).

### Safety and improved efficacy of diarrhea symptomatic treatment and reduced the antibiotic usage with high-dose and multi-strain *Bacillus* spore supplementation

Throughout the treatment period, we did not record any case of adverse reactions to the high dosages of LiveSpo DIA30 product, including vomiting, unpleasant digestive symptoms (temporary increase in gas and bloating), increased thirst, or symptoms of an allergic reaction. Clinical observations in the Dia30 group revealed progressive improvements in feeding tolerance, reduced irritability, and better physical comfort based on routine examinations. Moreover, there were no notable fluctuations in the body temperature of the children in both the control and Dia30 group (Fig. S1A-B) from day 1 to day 7 of measurement.

The probiotic supportive treatment demonstrated notable effectiveness in controlling a range of diarrhea symptoms (Table 2). Significant decrease in the number of patients having 6–7 stools per day (*p* = 0.0077), presence of fecal mucus (*p* = 0.0104), and diapered infant stool scale types 4-5B (*p* < 0.0001) at day 3 was observed in Dia30 group. At day 5, the numbers of patients having the three typical symptoms associated with diarrhea, including (i) the frequency of bowel movements of *≥* 3 times per day (3–5 stools a day), (ii) the presence of fecal mucus, and diapered infant stool scale types 4-5B, in Dia30 group were remarkably lower than those in the Control group (*p* = < 0.0001, < 0.0001, and = 0.0037, respectively).

The probiotic intervention yielded significant benefits. As shown in Table 3, Dia30 groups showed a significantly higher percentage of patients that were free of diarrhea symptoms (Dia30 70.83% vs. Control 20.41%, with an odds ratio (OR) of 9.47 and *p* < 0.0001). In fact, all children receiving LiveSpo DIA30 recovered within 10 days, while the control group required an additional 5 days of treatment. In terms of individual symptoms observed at day 5, the OR for frequency of bowel movements < 3 times per day in the Dia30 group vs. the Control group was 8.39 (*p* < 0.0001). Furthermore, it was observed that 75.00% patients in the Dia30 group had low or non-mucous stools compared to only 30.61% in the Control group, with OR = 6.80 (*p* < 0.0001). Lastly, the occurrence of soft stool types 2–3 (according to the Diapered classification) reached a higher rate in Dia30 group (79.17%) than in the Control group (51.02%), with OR of 3.65 (*p* = 0.0037).

**Table 3:** Resolution of symptoms and key subclinical indicators by day 5 of treatment period.

Characteristic	Control group (*N* = 49)	Dia30 group (*N* = 48)	OR	95% CI	*p* value
Free of all symptoms *n* (%)	10 (20.41)	34 (70.83)	9.47	3.75–22.5	**< 0.0001** ^**b**^
Stools a day of < 3 *n* (%)	11 (22.45)	34 (70.83)	8.39	3.41–19.67	**< 0.0001** ^**b**^
Absence of fecal mucus *n* (%)	15 (30.61)	36 (75.0)	6.80	2.86–16.87	**< 0.0001** ^**b**^
Diaper stool types 2–3 *n* (%)	25 (51.02)	38 (79.17)	3.65	1.5–9.31	**0.0037** ^**b**^
Erythrocyte negative and trace *n* (%)	44 (89.80)	47 (97.92)	5.34	0.67–64.08	0.2041^a^
Leukocyte negative and trace *n* (%)	43 (87.76)	48 (100)	14.49	0.79–264.9	0.0266^a^

^a^Fisher’s Exact test; ^b^Chi-Square test.

**Fig. 2:**
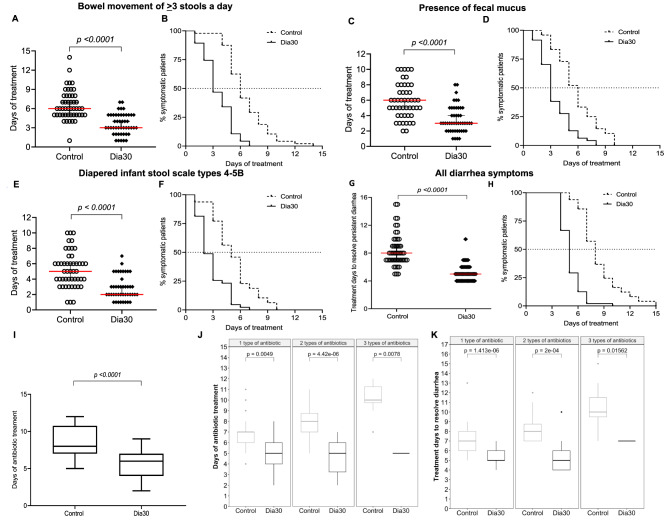
Days of treatment for observation of individual and all typical symptoms (**A**, **C**, **E**, **G**) of diarrhea and time-dependent percentage (%) of asymptomatic patients (**B**, **D**, **F**, **H**) in the Control (dashed lines) and Dia30 (solid lines) groups. Duration of antibiotic treatment prescribed for children with persistent diarrhea of the Control and Dia30 groups (**I**), and the duration of antibiotic treatment (**J**) and diarrhea treatment (**K**) across varying numbers of antibiotic types used for the Control and Dia30 groups. The difference between two distributions of independent groups was confirmed using the Mann-Whitney test. The significance level of all analyzes was set at the *p* < 0.05.

We further analyzed the treatment duration required to alleviate these three clinical symptoms. We also performed a Kaplan-Meier analysis to demonstrate the percentage of symptomatic patients over the course of treatment, allowing us to calculate the average *D*ay needed to *T*reat *50*% of total children (DT_50_) free from symptoms of persistent diarrhea. Dia30 group underwent significantly shorter treatment duration for all three symptoms i.e. having *≥* 3 bowel movements per day (DT_50_: Dia30 2.9 vs. Control 5.6 days), presence of fecal mucus (DT_50_: Dia30 2.6 vs. Control 5.1 days) and diaper stool types 4-5B (DT_50_: Dia30 2.0 vs. Control 4.6 days) (Fig. 2A-F). Notably, as shown in Fig. 2G-H, the overall treatment duration to completely recover diarrhea in the Dia30 group was almost halved, reduced to 5 days, compared to the 8 days required in the Control group, indicating a 1.60 -fold improvement in treatment efficacy.

Next, we were interested in evaluating whether the use of LiveSpo DIA30 reduces the time on antibiotic treatment in patients. Antibiotic therapy in children with persistent diarrhea were closely monitored by the physicians as described in Table S1and the Methods section. Antibiotic prescription was typically given at the beginning of treatment. However, throughout the entire course of treatments, antibiotics could be replaced or combined with new antibiotics depending on effectiveness as assessed during the initial 3 days of therapy. Therefore, the total number of antibiotics used in patients was accounted for at the end of the treatment. We found that the median duration of antibiotic use in the Dia30 group was approximately 6 days compared to up to 8 days in the Control group (*p* < 0.0001) (Fig. 2I). This is a 2-day reduction in antibiotic treatment duration for children with persistent diarrhea, corresponding to a 25% decrease in antibiotic usage. The benefits of shortening antibiotic treatment by Dia30 were consistently observed across different groups of patients prescribed with one antibiotic (7 days in Control vs. 5 days in Dia30), two antibiotics (8 days in Control vs. 5 days in Dia30), and three antibiotics (10 days in Control vs. 5 days in Dia30). In all cases, the differences were statistically significant, with *p*-values < 0.01 (Fig. 2J). Similarly, the shorten time needed for the complete resolution of diarrhea in the three patient groups correlated well with the reduced duration of antibiotic treatment (Fig. 2K). In patients prescribed one, two, or three types of antibiotics, the Dia30 group demonstrated a significantly shorter duration of recovery from diarrhea compared to the Control group, with differences of 2 days (7 days in Control vs. 5 days in Dia30), 3 days (8 days in Control vs. 5 days in Dia30), and 3 days (10 days in Control vs. 7 days in Dia30), respectively (all *p*-values < 0.02).

### Reduced fecal leukocytes alongside modulation of blood cytokines and fecal IgA through high-dose, multi-strain *Bacillus* spore supplementation

To evaluate the disease status, we measured three routine fecal biochemical parameters to examine the presence of erythrocytes and leukocytes (categorized as mild, moderate, and severe levels), and stool pH of *≤* 5.5. We found two notable differences in the leukocyte indicator in the Dia30 group when compared to the Control group (Table 2). Firstly, by day 3, while over 50% of patients the Control group (*n* = 25) still exhibited mild-to-severe leukocyte positivity, there was only 20.83% of patients in the Dia30 group presented with similar indicators (*n* = 10). Secondly, after 5 days of treatment, while mild-to-severe leukocyte positivity was still observed in 6 cases in the Control group (12.24%), the positivity as completely cleared in the Dia30 group (0%). The other two parameters including fecal erythrocytes and pH *≤* 5.5 did not show statistically significant differences between the two groups either at day 3 or day 5. In addition, when examining the two primary indices employed for evaluating recovery from diarrhea e.g. erythrocytes and leukocytes in stool—set at negative (-) or trace positive clinical thresholds, they were observed in the Dia30 group, with an odds ratio (OR) (95% CI) of 5.34 (0.67–64.08) and 14.49 (0.79–264.9), respectively, compared to the Control group (Table 3).

**Fig. 3:**
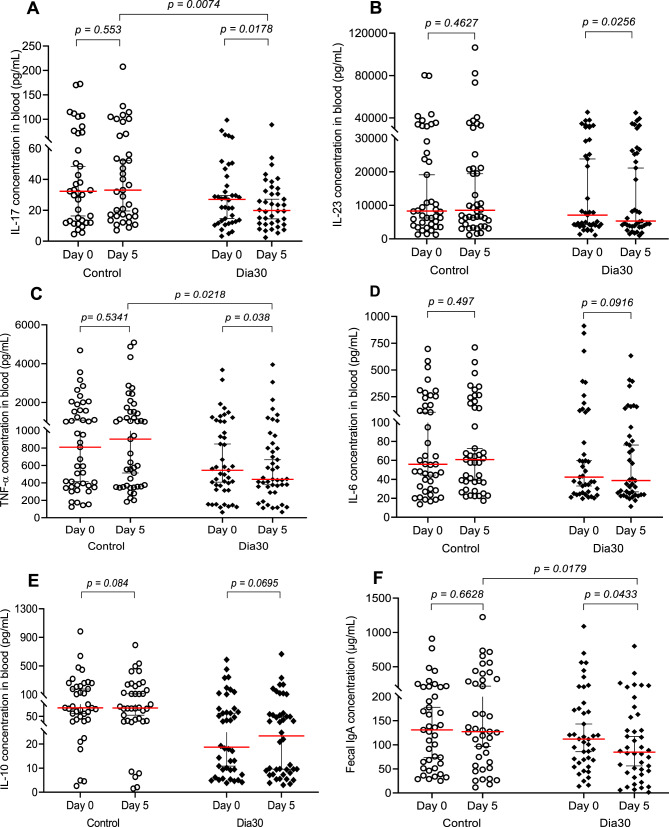
Pro- and anti-inflammatory cytokines levels (pg/mL) in blood samples and IgA levels (µg/mL) in stool samples of Control and Dia30 groups at day 5 compared to day 0. The Wilcoxon signed-rank test was used to calculate the median differences in IL-17 (**A**), IL-23 (**B**), TNF-α (**C**), and IL-6 (**D**) pro-inflammatory cytokine, anti-inflammatory IL-10 (**E**) cytokine, and IgA (**F**) levels at day 0 and day 5 in each group. The Mann-Whitney test was used to compared cytokine and IgA concentrations (**A**–**F**) between the two independent groups. Only samples with measurable cytokine and IgA concentrations at day 0 were included in the statistical analysis. 95% CI for median in each group and the median difference between the two groups were shown in Fig. 3. The significance level of all analyzes was set at the *p* < 0.05.

To investigate the immunomodulatory effects of the LiveSpo DIA30 on the intestinal immune system in persistent diarrhea, we examined changes of representative pro/anti-inflammatory cytokines in the blood and IgA levels in fecal samples between day 0 and day 5. The time points were chosen to capture the period when the clinical symptoms displayed the most noticeable improvements. We observed that over the course of 5 days, LiveSpo DIA30 treatment led to a significant decrease in IL-17 and IL-23 (by 26.62% and 25.13%, with *p* values of 0.0178 and 0.0256, respectively), a 19.09% (*p* = 0.038) reduction in TNF-α signaling and a decreasing trend in IL-6 (8.54%; *p* = 0.0916) (Fig. 3A-D). The data indicated an overall suppression of the Th17 pathway. In contrast, the Control group did not exhibit significant changes in these pro-inflammatory cytokines, with very modest increases in IL-17, IL-23, TNF-α, and IL-6 (*p* values > 0.1) (Fig. 3A-D). Consistently, the anti-inflammatory IL-10 showed a 24.94% increase in the Dia30 group (*p* = 0.0695), while this value slightly decreased by 0.37% in the Control (*p* = 0.084) (Fig. 3E). In addition, we only noted a significant decrease in IgA levels in the group of patients treated with probiotics (24.24%; *p* = 0.0433 in Dia30 vs. 2.85%, *p* = 0.6628 in Control) (Fig. 3F). When comparing the concentrations of cytokines and IgA between Control and Dia30 before treatment, we observed that most of the 5/6 indices did not show statistically significant differences. However, by day 5, there was notable reduction in IL-17 and TNF-α pro-inflammatory cytokine levels, and IgA level in the Dia30 group (Fig. 3A, C, and F). Difference in IL-10 levels was already evident at day 0, making the differences observed on day 5 not suitable for analysis (Fig. 3E). The IL-6 and IL-23 levels of Dia30 at day 5 was reduced about 1.6-fold compared to the Control group, but the differences were not significant (*p* > 0.05). (Fig. 3B and D). Overall, the LiveSpo DIA30 treament resulted in a significant reduction in fecal leukocyte positivity, pro-inflammatory blood cytokines such as IL-17, TNF-α, and fecal sIgA in comparison to the Control group.

### Restoration of alpha diversity and density of phylum, family, genus, and beneficial species of gut microbiota by high-dose, multi-strain B*acillus* spore supplementation

**Fig. 4:**
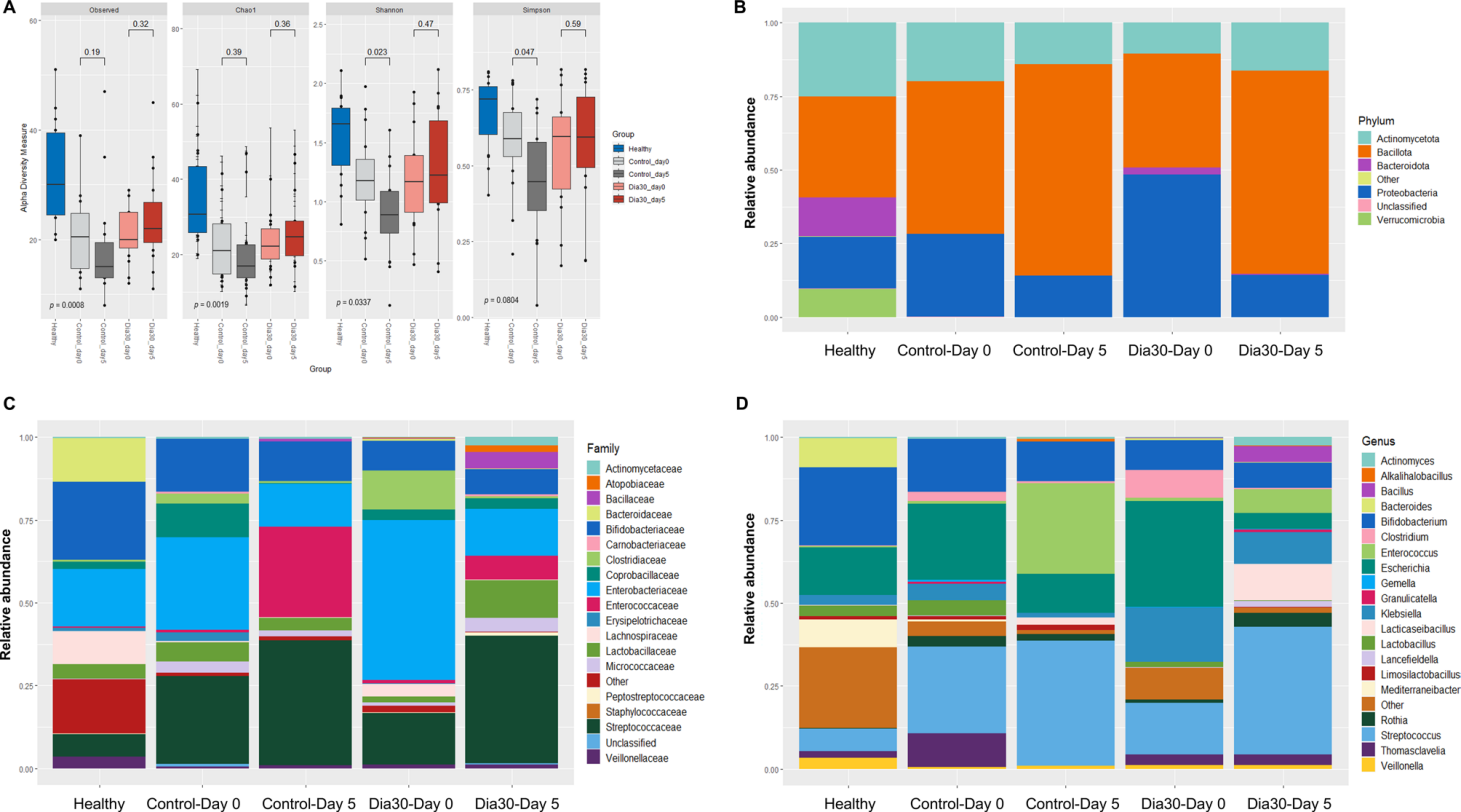
Alpha diversity (**A**), distribution of major phyla (**B**), families (**C**), and genera (**D**) of 16S rRNA microbiota in stool samples were compared between healthy children and the Control and Dia30 groups on day 5 versus day 0. The Kruskal-Wallis test was employed for both pairwise and overall comparisons in alpha diversity (Observed OTU, Chao1, Shannon, Simpson) for overall and in-pair comparisons. The significance level of all analyzes was set at the *p* < 0.05.

The gut microbiota plays a vital role in promoting and maintaining guthealth. Therefore, we evaluated the changes in gut microbiota in children with persistent diarrhea undergoing probiotic treatment on day 5 vs. day 0, with reference to the gut microbiota in healthy children. As expected from diarrhea-induced inferior richness and diversity of microbial species, alpha diversity indices of the diarrhea groups before treatment were lower than those of the healthy group, with corresponding reductions from 30,00 to 20.25 for Observed OTU, from 30.75 to 21.68 for Chao1, from 1.66 to 1.17 for Shannon, from 0.72 to 0.59 for Simpson index (Fig. 4A). In the Control group at day 5, these indices decreased from 20.50 to 15.00 for Observed OTU, from 21.10 to 17.00 for Chao1, from 1.18 to 0.89 for Shannon, and from 0.59 to 0.45 for Simpson diversity index, with statistically significant difference for Shannon and Simpson values (*p* < 0.05). In contrast, the Dia30 group at day 5 exhibited an increase in the three indices, including Observed OTU (from 20.00 to 22.00), Chao1 (from 22.25 to 24.63), and Shannon (from 1.17 to 1.23), however all the differences were not statistically significant (*p* > 0.05) (Fig. 4A). PCoA analysis of beta diversity showed overlap between all tested samples. The results indicated that there was no marked difference in the composition of gut microbiota between the diarrhea vs. healthy groups either before or after probiotic treatment (Fig. S2).

To explore improvements in gut microbiota diversity in children with diarrhea treated with probiotics, we analyzed 16 S rRNA microbial components in stool samples from Control and Dia30 groups (*n* = 16). In parallel, we also benchmarked the results with a reference group of healthy children (*n* = 15). As shown in Fig. 4B, we found that the overall composition of the gut microbiota in both healthy children and those with persistent diarrhea is predominantly influenced by four main bacterial phyla: Bacillota (34.3%), Actinomycetota (25.0%), Proteobacteria (17.6%), and Bacteroidota (13.2%). Before treatment, the diarrhea groups showed abnormal microbial phyla distribution, with increased densities of Proteobacteria (38.2%) and Bacillota (45.3%), while Actinomycetota and Bacteroidota, which are dominant in healthy children, decreased to approximately 15.2% and 1.2%, respectively. At day 5, we observed a 1.4-fold increase in the density of the Bacillota in the Control group and a 1.8-fold increase in the Dia30 group. Additionally, there was a 1.5-fold increase in the density of the Actinomycetota in the Dia30 group, whereas a 1.4-fold decrease was observed in the Control group. In terms of the Proteobacteria, its density decreased by 2-fold and 3.4-fold, respectively, in the Control and Dia30 groups by day 5, approaching the levels observed in healthy children. None of the diarrhea groups fully restored the bacteria belonging to the Bacteroidota at day 5.

We also evaluated 18 families with the highest microbial density (Fig. 4C). The healthy group exhibited large proportions of families of Bifidobacteriaceae (23.6%), Enterobacteriaceae (17.32%), Bacteroidaceae (13.17%), Streptococcaceae (6.78%), and Lactobacillaceae (4.45%), and small representation of Clostridiaceae (0.6%) and Enterococcaceae (0.4%). On the other hand, diarrhea group exhibited a markly different distribution, with the Enterobacteriaceae having the highest density (38.4%), followed by Streptococcaceae (20.95%). Meanwhile, Bifidobacteriaceae, Bacteroidaceae, Lactobacillaceae, and Staphylococcaceae exhibited lower densities, with corresponding values of 12.51%, 0.28%, 3.74%, and 0.03% respectively. The density of Clostridiaceae family experienced a significant increase, reaching 7.25%. At day 5, both the Control and Dia30 groups showed significant redistribution of microorganism families. Enterobacteriaceae and Clostridiaceae family were reduced by 2.14- and 4.17-fold in the Control group and by 3.40- and 24.3-fold in the Dia30 groups. Bifidobacteriaceae and Bacteroidaceae experienced a decrease of 1.34-fold and 15.23-fold in Control group and only 1.19-fold and 3.47-fold in probiotic treated group. On the other hand, the density of Enterococcaceae increased by 38.80-fold and Streptococcaceae increased by 1.42-fold in the Control group, while in the Dia30 group, they increased by 6.88-fold and 2.49-fold, respectively. Notably, density of Lactobacillaceae increased by 6.62-fold only in the Dia30, while it decreased in the Control group by 1.47-fold. It is also noteworthy that there was an increase in the Atopobiaceae only in the Dia30 group, with a density of 2.0%, whereas it was either absent or below the detection threshold in the Control group.

In addition, we conducted distribution-analysis of 20 common genera with the highest density (Fig. 4D). In the healthy group, *Bifidobacteria* exhibited the highest density (23.6%), followed by *Escherichia* (14.31%), *Bacteroides* (8.75%), and *Streptococcus* (6.78%). Other common genera in the gut intestine with lower density included *Lactobacillus* (3.13%), *Klebsiella* (2.87%), *Clostridium* (0.23%), *Enterococcus* (0.4%), *Lacticaseibacillus* (0.29%). In contrast, in children with diarrhea, the distribution of *Bifidobacteria* and *Bacteroides* was lower, accounting for only 12.51% and 0.26%, respectively, while *Escherichia* and *Streptococcus* were the dominant groups, accounting for 27.51% and 20.73%, respectively. Furthermore, the *Clostridium* and *Klebsiella* became more abundant, accounting for up to 5.53% and 10.62%, which was 24-fold and 3.7-fold higher than those of the reference healthy group, respectively. At day 5, both the Control and Dia30 groups clearly showed a decrease in the density of *Escherichia*,* Klebsiella*, and *Clostridium* genera that are known to contain harmful species. Notably, *Escherichia* decreased by 6.82-fold in the Dia30 group, whereas it decreased by only 1.97 folds in the Control group; and *Clostridium* decreased by 17.79 folds in the Dia30 group, while the Control group showed a lower decrease by 4.09 folds. However, *Klebsiella* exhibited a more modest reduction of 1.71 folds in the Dia30 group compared to 3.57 folds in the Control group. Concerning genera known to contain beneficial species, we observed a decrease in the density of the *Bifidobacterium* and *Lactobacillus* by 1.34 folds and 719 folds in the Control group, whereas they reduced to a lesser extent by 1.19 folds and 28.19 folds in the Dia30 group. Remarkably, there was a significant increase in the density of the *Lacticaseibacillus*, up to 11.04% in the Dia30 group compared to 2.15% in the Control group on day 5, while it remained low in the healthy children group (0.29%) and undetectable in the diarrhea group before treatment. Interestingly, the *Thomasclavelia* genus significantly declined in the Control group by 120.8 folds while its density remained unchanged in the Dia30 group.

**Fig. 5:**
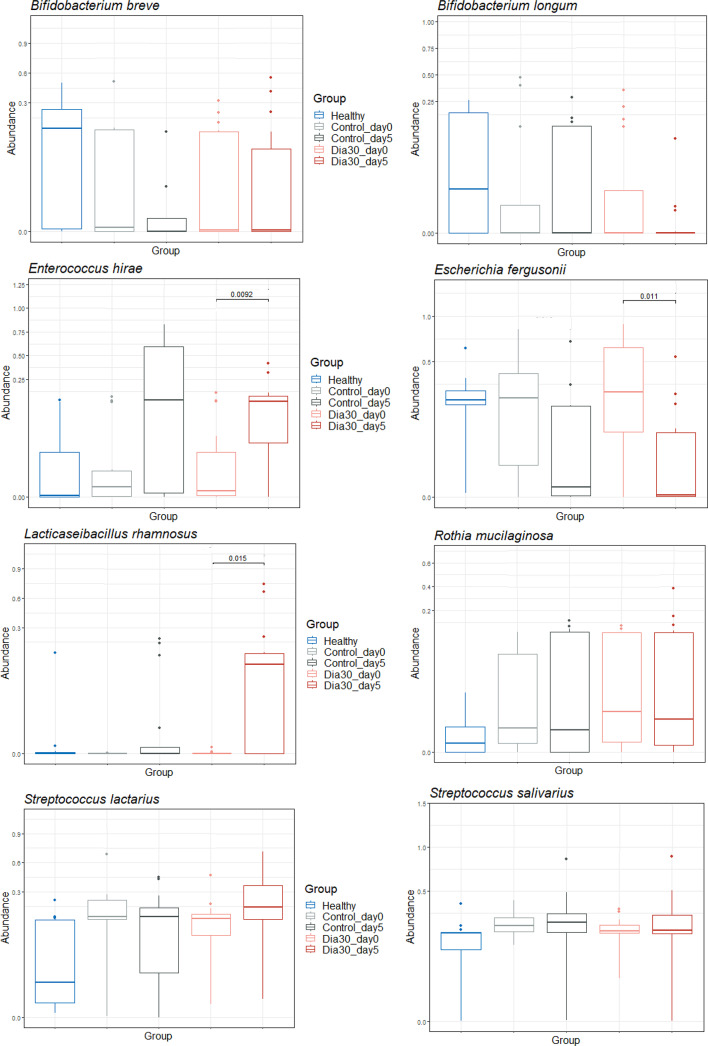
Comparison of the top-eight abundant native species of 16S rRNA microbiota in stool samples from healthy children, Control and Dia30 groups at days 0 and 5. The Wilcoxon test was used to assess differences in bacterial abundance between these time points. The Mann-Whitney test was used for comparing the difference between two independent groups. The significance level of all analyzes was set at the *p* < 0.05.

Finally, we selected top-8 native species with high relative abundance in the gut for further analysis to determine the impact of probiotic intervention (Fig. 5). We observed both increases and decreases in the density of a range of species in the Dia30 group compared to the Control group at day 5. The results are in agreement with the findings from the analysis of phyla, families, and genera. Following 5 days of treatment, the Dia30 group demonstrated a preservation of *Bifidobacterium breve* species density with slightly 1.36-fold decrease, while the Control group exhibited much more substantial 14.19-fold decrease, compared to day 0. Additionally, the density of the harmful species *Escherichia fergusoni* significantly decreased by 682.8 folds in the Dia30 group (*p* = 0.011), whereas this species non-significantly decreased to a much lesser extent in the Control group (89.9-fold; *p* = 0.079). On the other hand, *Enterococcus hirae* increased by 29.49 folds in the Control group, and 25.76 folds in the Dia30, but the increase was only significant in the Dia30 group (*p* = 0.096 for Control and *p* = 0.0092 for Dia30). Notably, the beneficial *Lacticaseibacillus rhamnosus* was almost absent in the diarrhea group at day 0. However, at day 5, this species exhibited a significant density of 0.91% in Dia30 (*p* = 0.015) whereas it remained below the detectable levels in both Healthy group and the Control group. Similarly, the *Streptococcus lactarius* at day 5 also increased compared to day 0, with a higher increase in the Dia30 group (5.22-fold), whereas it remained almost unchanged in the Control group (1.06-fold). For the remaining three species including *B. longum*,* Rothia mucilaginosa*, and *S. salivarius*, there was no notable change between the Dia30 group and the Control group on day 5.

Overall, these results support the restoration of alpha diversity, the establishment of phyla, families, genera, and beneficial species, and the reduction of harmful species associated with gut health following probiotic treatments in pediatric patients with diarrhea.

## Discussion

*Bacillus* spore probiotics have been extensively studied for the treatment of gut dysbiosis at the conventional dosage of 2–4 billion CFU daily^21–23,26^. In our previous clinical study on children with persistent diarrhea, we used a high dosage of single-strain *B. clausii* probiotics (8–12 billion CFU daily) as supportive treatment, resulting in a 1.6-fold increase in efficacy and a 3-day reduction in recovery time^24^. Here, we reported the first study on the safety and efficacy of multi-strain *Bacillus* spore probiotics (20–30 billion CFU daily) for managing persistent diarrhea in children aged 3 to 24 months. Our safety assessment found the treatment well-tolerated with no adverse events. The results demonstrated a significant reduction in both symptomatic and overall treatment duration, aligning with our previous single-strain *B. clausii* study and other probiotic trials, showing improved bowel movements and stool consistency^20–23,25−28^. While the outcomes indicating a reduction in bowel movement frequency in these studies are noteworthy, odds ratios with confidence intervals have not been evaluated to reflect the level of certainty. Thus far, the treatment regimen tested in the current study exhibited superior efficacy. The benefits of reducing the duration of resolution of persistent diarrhea are in multiple folds, not only improving children’s health but also alleviating parental anxiety, reduces caregiving time, and lowering therapy and hospitalization costs. This is particularly significant considering that children under two years old in low-income countries typically experience an average of three episodes of diarrhea each year, and given the increasing incidence of diarrhea cases due to post COVID-19 infections.

The intestinal mucus layer is crucial for maintaining intestinal health by protecting against microorganisms, serving as a substrate for bacterial growth, and supplying binding sites, nutrients, and structure. It acts as a vital defense mechanism against bacteria^29,30^. While trace amounts of mucus in stools are normal, an excessive presence may indicate a digestive disorder. Our study revealed a faster reduction in leukocytes and mucus in stools for the Dia30 group, suggesting that the multi-strain *Bacillus* spores may enhance the mucosal barrier, driven by the gut microbiome^30^. While we could not directly measure IgA in the children’s mucus layer, the observed reduction in fecal mucus, leukocytes, and IgA levels suggests recovery and increased IgA on the intestinal epithelium in patients using LiveSpo DIA30. Cytokine expression and CTL proliferation depend on T-helper responses. Diarrhea in children is marked by intense neutrophilia and overreacted release of pro-inflammatory cytokines like TNF-α, IL-6, and IL-8 due to infections or microbiota imbalance. Additionally, the gut microbiota acts as a potent immunoregulator, with the IL-23/IL-17 axis playing a central role^29,31^. Notably, as the secretion of IgA from intestinal mucous is known to be promoted by IL-17, the observed Th17 depression in patients receiving Dia30 probiotics may partially contribute to the significant reduction in IgA levels detected in their feces. Beside the IL-17 pathway, we also observed the modulation of other cytokines like IL-6, TNF-α, which are potential biomarkers for gut inflammation. Therefore, this study supports the notion that while cell mediated immunity does not specially prevent microbial infection, it can reduce the gut inflammation and reduce the diarrhea symptoms. Our findings align with a decrease in IL-17, TNF-α, and IL-6 reported in a recent clinical trial using the *B. clausii* UBBC-07 strain in inflammatory bowel disease^26^.

The gut microbiota plays a crucial role in influencing gut health, including metabolism, nutrient exchange, and the immune system. It closely interacts with various types of immune cells, notably Th17 cells, regulatory T cells, and antigen-specific B cells for the intestine^12,32–34^. While complete restoration to healthy levels was not achieved within the study period, the Dia30 group demonstrated significant trends toward healthy microbiota profiles. Specifically, the Dia30 group showed significant recovery in OTU, Chao1, Shannon, and Simpson indices compared to the Control, indicating that LiveSpo DIA30 significantly recovered gut microbiota diversity in persistent diarrhea patients. This aligns with the markedly improved clinical symptoms observed on day 5 of treatment. Our findings align with previous studies showing that probiotics improve gut microbiota diversity, particularly in children. For instance, Castro-Mejía et al. (2020) reported enhanced Shannon and Chao1 indices in malnourished children^35^while Korpela et al. (2018) observed similar increased Shannon and Simpson indices in cesarean-born infants^36^. These studies support our conclusion that probiotics can increase diversity, though full recovery may require a longer intervention period. We observed a remarkable reduction in *E. fergusoni* density in the Dia30 group on day 5, contributing to decreased densities of the *Escherichia* genus, Enterobacteriaceae family, and Proteobacteria phylum. Changes in growth rates of certain Gram (-) pathogens within these groups have been suggested as catalysts disrupting gut microbiota balance and causing gastrointestinal disorders^37^. Several studies have also reported the ability of *Bacillus* probiotics in inhibiting pathogenic gut bacteria species through competition for adhesion sites on the intestinal mucosa, nutrient competition, and secretion of antibacterial lipopeptides targeting gastrointestinal pathogens^38,39^. Thus, the effect of LiveSpo DIA30 in reducing the density of *Escherichia* genus may play a beneficial role in restoring the dysbiosis of the gut microbiota. Some antibiotics have been reported to strongly inhibit beneficial species of the *Bacteroide*s and *Bifidobacteria* genera, leading to gut microbiota imbalance^40^. Therefore, the positive influence on the mild reduction of *Bifidobacterium* bacteria by LiveSpo DIA30, is also a favorable indication for reducing the growth of potential pathogens that are still unknown. The beneficial *L. rhamnosus* is a well-known gut-origin probiotic, commonly supplemented daily for improving the gut immune and preventing intestinal diseases^41,42^. Notably, the beneficial bacterium *L. rhamnosus*, increased to a greater extent in the Dia30 group than in the Control group. This could be explained by the presence of the three *Bacillus* strains which secret amylase and protease at high levels, thereby maximizing the hydrolysis of carbohydrate and protein sources into readily usable simple sugars and amino acids for the growth of *L. rhamnosus.* Consequently, the secondary compounds, such as short chain fatty acids (SCFA) and lactic acids, produced by *Bacillus* and *Lacticaseibacillus* species may also contribute to enhancing the effectiveness of diarrhea reduction and immune system regulation^38,41^. In addition, the increased density of *S. lactarius*, predominant species found in the oral cavity or breast milk of breastfeeding infants^43^may potentially contribute to the rapid rebalancing of the gut microbiota facilitated by the combined *Bacillus* spores. Alongside this, there was an increase in the *Enterococcus* genus in both groups, which may be closely related to antibiotic treatment, especially Ceftriaxone^40,44^. The species *E. hirae*, observed to significantly increase in the Dia30 group compared to the Control group, is a beneficial bacterium known for its resistance to pathogenic species within the same *Enterococcus* genus, such as antibiotic-resistant strains like *E. faecium* and *E. faecalis*^45^. Interestingly, increased density of Atopobiaceae bacteria family is observed only in the Dia30 group. These bacteria, recognized as potentially beneficial in mammalian digestive systems, produce SCFA, beneficial lactates, and bile salt hydrolase (BSH) enzymes with antibacterial and cholesterol-lowering properties, making them a target for future biotherapeutic research^46–48^. Finally, we observed a significant presence of *B. subtilis*, which may result from the oral administration of LiveSpo DIA30, with *B. subtilis* being particularly capable of germination and rapid growth compared to the other two *B. clausii* and *B. coagulans* species. This observation aligns with the properties of the *Bacillus* strains in LiveSpo DIA30. These strains exist as spores, naturally resistant to harsh gastrointestinal conditions and antibiotics. Administering probiotics at least 2 h apart from antibiotics likely minimized their exposure to antibiotics while maintaining the spores’ ability to germinate when conditions become favourable. Taken together, the microbiota results indicate a significant improvement in the gut microbiota diversity of children with persistent diarrhea upon probiotic supplementation. This improvement is consistent with the observed decrease in IL-17 and IL-23, IL-6, and TNF-α in Dia30 groups and provides scientific evidence for elucidating the mechanism of probiotics in regulating gut immunity through the IL-23/IL-17 axis and through balancing the Th1/Th2 immune pathways. A limitation of our study is the lack of statistically significant differences in IL-6 and IL-10 values between day 5 and day 0 in the Dia30 group and in IL-23 values between the two groups on day 5 (*p* > 0.05) due to small sample size. Due to budget and resource constraints, we were limited to one high-dose probiotic therapy (20–30 billion CFU daily) and could not investigate dose-dependent effects; additionally, only about one-third of the samples were analyzed for the 16S RNA metagenome, which may have contributed to the lack of statistical significance observed in some taxonomy classification indices.

In conclusion, high-dosage treatment with combined *Bacillus* spores was well-tolerated, with no adverse events. The Dia30 group had a 3-day shorter recovery period, 1.60 -fold increased efficacy, and a 9.47-fold higher chance of resolving diarrhea after 5 days. High-dose LiveSpo DIA30 reduced antibiotic treatment time and medical expenses by 25%. Significant reductions in elevated blood pro-inflammatory cytokines, including IL-17 (26.62%), IL-23 (25.13%), and TNF-α (19.09%), as well as in fecal sIgA (24.24%), were observed in the Dia30 group. Improvements in clinical and subclinical indicators can be attributed to the role of *Bacillus* spores which balanced the gut microbiota by increasing beneficial species like *L. rhamnosus* and reducing harmful ones like *E. fergusoni*. This probiotic therapy shows significant promise for treating diarrhea, particularly in low- and middle-income countries.

## Methods

### Materials

The food supplement LiveSpo DIA30, produced by LiveSpo Pharma in Hanoi, Vietnam, is a liquid water suspension containing *B. subtilis* ANA48, *B. clausii* ANA39, and *B. coagulans* ANA40 spores at a concentration *≥* 5 × 10^9^ colony-forming units (CFU) per 5 mL ampoule. This product was manufactured in accordance with Good Manufacturing Practice (GMP) standards approved by the Ministry of Health, Vietnam, with certificates numbered 18/2021/ATTP/CNG-GMP for good manufacturing practice and VICB 7831.6-A for Hazard Analysis and Critical Control Points (HACCP). Registration of the product was 6547/2019/DKSP. Prior to manufacturing and conducting clinical studies, the strains underwent rigorous in-vitro physiological and biochemical testing to guarantee their safety and probiotic properties. The summarized data include (i) microbial and biochemical characterization (Table S2); (ii) antibiotics susceptibility (Table S3); (iii) 16 S rRNA sequencing analysis (Fig. S3-S5) with the following accession numbers: PQ425921 for *B. subtilis* ANA48, MT275656 for *B. clausii* ANA39, and MT734108 for *B. coagulans* ANA40; and (iv) sequence analysis of toxic genes by PCR and whole genome sequencing of *B. subtilis* ANA48, *B. clausii* ANA39, and *B. coagulans* ANA40 (Table S4). Acute and sub-acute toxicity studies had been conducted on mice and rabbits, administering dosages 20-fold and 3-fold higher than typical human doses. These investigations were conducted by the Vietnam National Drug Quality Control Institute, confirming the non-toxic nature of LiveSpo DIA30. Notably, the taste, odor, color, and turbidity of LiveSpo DIA30 and the control product (RO) are indistinguishable due to the opaque plastic ampoules. The control and intervention products were designated as codes A and B, respectively, with this information kept confidential from parents of children, nurses, doctors, and investigators.

### Ethical issues

This research received approval from the Ethics Committee in Medical Research of the Vietnam National Children’s Hospital, as per Decision 1077/BVNTU-HDDD dated June 1, 2022. The study adhered to ethical principles in accordance with the Helsinki Declaration, ICH Good Clinical Practice (GCP) guidelines, and the regulations and standards on human subject research set forth by the Vietnam Ministry of Health. All parents of pediatric patients who participated in the study were fully informed about the study and provided their consent by signing the informed consent form. Furthermore, participants had the option to exit the study at any time if they wished to do so. The study was also registered with ClinicalTrials.gov under Identifier No: NCT05812820, 14/4/2023.

### Study design and patient collection

This study was a double-blind,randomized, controlled trial involving two groups: a Control group receiving reverse osmosis (RO) water and an experimental group (referred to as the “Dia30” group) used the probiotics LiveSpo DIA30. The trial followed the CONSORT 2010 guidelines for reporting parallel group randomized trials. The study extended over 8-month period, from April 15, 2023 to December 25 2023, and included pediatric patients of both genders who were experiencing persistent diarrhea at the Department of Gastroenterology, Vietnam National Children’s Hospital. The sample size of children with persistent diarrhea was calculated based on preceding data successfully obtained with the single-strain *B. clausii* spore probiotics (LiveSpo CLAUSY) with a hypothesis that LiveSpo DIA30 would alleviate persistent diarrhea symptoms by about 30% more effectively (α = 0.05; power level = 0.9). This was inferred from the expectation that 90% of patients in the Dia30 group would be symptom free at day 5–10 of intervention, compared to 60% of patients in the Control group^24^. These proportions, along with the two-sided confidence level (1-α) of 95% and the 90% chance of detecting (the power 1-$\:\beta\:$), were used to calculate the minimum sample size for each group based on Kelsey formula as follow: n_kelsey_ = $\:\frac{{\left({Z}_{\alpha\:}+\:{Z}_{\beta\:}\right)}^{2\:}p(1-p)\left(1+k\right)}{{k\left({p}_{0}-{p}_{1}\right)}^{2}}$, where, $\:k=1$ is the ratio of Controls to Cases; α = 0.05; Z_α_ = 1.96; β = 0.1; Z_β_ = 1.28; $\:p=\frac{{p}_{0}+\:k{p}_{1}}{1+k}=\frac{{p}_{0}+{p}_{1}}{2}$; $\:{p}_{0}=\text{0.6};\:\:{p}_{1}=\text{0.9}.$ The estimated required minimum sample size at the end of intervention for two groups was 88, equivalent to 44 for each group. In fact, a total of 126 patients with persistent diarrhea, who were admitted to the Department of Gastrointestinal at Vietnam National Children’s Hospital, were screened for eligibility. Out of these, 100 eligible participants (*n* = 50 per group) were randomly assigned to either the Control or Dia30 group to minimize the risk of patient dropouts (about 20%) during follow-up treatment. Eligible participants were randomly assigned a study ID number from 1 to 100 to the Control and the Dia30 groups. The permuted block randomization technique was applied, in which patients were allocated to blocks of random size, with a size of 2. For each block, patients were randomly allocated to Dia30 or Control groups. The randomization code was generated electronically by an IT specialist from the Pharmaceutical Department of Vietnam National Children’s Hospital, utilizing the Excel RAND function (Microsoft, WA, US). Subsequently, the pharmacist responsible for dispensing the study products had access to the corresponding key. The labels A and B were assigned to the Control and the Dia30 groups, respectively, with this information being kept confidential from parents of children, nurses, doctors, and investigators. The flow charge of study was presented in Fig. 1.

Inclusion criteria were:

1. Patients aged between 3 and 24 months.
2. Patients experiencing loose stools or abnormal water on ≥ 3 times/day for a duration of 14 to 30 days without symptom improvement, require hospitalization in the Department of Gastroenterology at the National Children’s Hospital in Vietnam for internal care treatment.
3. The child’s parents or caregivers understand the content of the interview questions, consent to participation, and adhere to the study protocol.

Exclusion criteria included:

1. Patients with any systemic illness other than diarrhea upon admission.
2. Patients experiencing any systemic complications during treatment.
3. Patients diagnosed with inflammatory bowel diseases (such as Crohn’s disease, ulcerative colitis), tuberculosis, and HIV.
4. Patients with a history of preterm delivery; children suffering from severe malnutrition and/or edema.
5. Patients who have been treated with spore-forming probiotics containing one of the following species: *B. subtilis*, *B. clausii*, or *B. coagulans* prior to study participation, and/or have been detected with one of these species in stool samples before treatment initiation using real-time PCR.
6. Parents or guardians directly responsible for the child’s care refusing to participate or withdrawing from the study during its course.

### Questionnaires, treatment procedures, and clinical observation

Parents of the patients were requested to provide the following details about their children: full name, gender, age, obstetric history, vaccination history, history of antibiotic use, duration of diarrhea, and any underlying medical conditions. Nurses underwent training to administer a dosage of 2 ampoules (either a placebo or probiotics) per administration, three times a day (6 ampoules daily) for the initial 3 days, followed by two times a day (4 ampoules daily) for the subsequent days of treatment. The placebo or LiveSpo DIA30 was administered using the ampoule dropper after gentle shaking, timed between antibiotic doses. Each administration was recorded, and infant acceptance was closely monitored. The treatment duration varied depending on the severity of the illness and the patient’s response to the treatment protocol. It was calculated as the day the patient recovers from all symptoms, typically ranging from 5 to 10 days. The placebo or LiveSpo DIA30 was prescribed for patients until their discharge from hospitalization, typically extending treatment time by an additional 2 days to ensure complete recovery and prevent any recurrence of the disease before hospital discharge. With the exception of patients who withdrew from the study, all patients were discharged in the afternoon of day 5 as earliest for the purpose of follow-up monitoring symptomatic clinical and sub-clinical indicators, even if all symptoms had resolved earlier.

The standard of care for patients with persistent diarrhea was tailored to each child’s age and clinical presentation. This protocol encompassed several key interventions, as summarized in Table S1. The treatment approach involved addressing dehydration through the administration of oral rehydration solution (ORS), zinc supplementation, nutrient therapy, and antibiotics for bacterial infections. The administration of ORS adhered to the guidelines set by the World Health Organization (WHO). For patients without signs of dehydration, ORS was utilized to maintain hydration by compensating for stool losses. In cases of minimal stool output, ORS might be deemed unnecessary. The volume of ORS usage depends on the child’s age and the degree of dehydration. Children under 2 years old without signs of dehydration were given 50–100 mL after each episode of diarrhea. Those with some dehydration received 75 mL/kg body weight every 4 h, necessitating supervision and frequent reassessment. No participants exhibited severe dehydration, eliminating the need for intravenous fluid therapy. While individual ORS volumes were not specifically recorded, the protocol ensured that all patients were adequately hydrated prior to randomization and intervention. Zinc supplementation followed WHO recommendations, with a dosage of 10 mg/day for 10–14 days for children under 6 months and 20 mg/day for older children. Nutritional therapy adhered to the hospital’s Nutrition Department guidelines, in line with recommendations from the Vietnamese Ministry of Health. This involved ensuring appropriate nutrition for patients, with breastfeeding advised for infants under 6 months. In cases of insufficient breast milk, lactose-free commercial formula was provided for the initial 5 days, followed by commercial digested formula if the lactose-free formula proves ineffective in improving diarrhea symptoms within the first 5 days. For children over 6 months, breastfeeding continued, and an optimal mixed diet (lactose-free milk for initial 5 days and digested milk for following days with reduced starch, replacing milk protein with chicken/egg) was supplemented. Total daily calorie intake aimed for approximately 100 kcal/kg, with a protein intake of 2–3 g/kg. Antibiotics were administered when intestinal bacterial infection was suspected,

determined by the presence of enteric bacterial pathogens or leukocytes and/or erythrocytes in stool samples. In case of positive pathogenic bacteria, antibiotic susceptibility tests were conducted, and patients received appropriate treatment accordingly. In cases where the initial single antibiotic regimen was ineffective within the first 3 days, adjustments or combinations with new antibiotics were prescribed based on the identification of underlying causes, influencing factors found at that time, or the hospital’s standard protocol for selecting alternative antibiotics. These practices aligned with the hospital’s protocols, Vietnamese Ministry of Health recommendations, and guidelines from the Infectious Diseases Society of America (IDSA).

Average weight was measured at different time points (days 0, 3, and 5), while height was measured at day 0, as it remained unchanged due to the relatively short treatment duration for diarrhea in children in this study. Weight for age Z-scores and Height for age Z-scores were assessed using the WHO child growth standards.

For primary outcomes, patients were monitored daily for common diarrhea symptoms, which included bowel movements exceeding three stools per day, the presence of fecal mucus, and the characteristics of diapered stool based on the Diapered Infant Stool Scale (types: 3-soft but separable; 4-mucosy, stringy, more fluid than soft; 5 A-watery with curds/solids; and 5B-watery without curds/solids) until their discharge.

The patients’ health status was monitored by doctors and nurses and then documented in their medical records. Parents of the children received guidance from medical professionals to undertake the following tasks: (1) keep a record of their children’s daily bowel movements; (2) use a mobile phone to capture photos of their child’s stools, assessing for the presence of fecal mucus and categorizing stool types based on the Diapered Infant Stool Scale atlas and reference photos displayed at the head of the bed. Throughout the entire treatment period, parents diligently counted the frequency of stools and took stool photos. Subsequently, they shared this data directly with the responsible doctors and nurses through discussions held every 8 a.m and 4 p.m.

Concerning adverse reactions, unpleasant digestive symptoms, increased thirst, and potential allergic reactions (if they occurred) was assessed by doctors. Assigned nurses directly measured body temperatures, while any vomiting symptoms were monitored by parents and promptly reported to the responsible doctors or nurses.

### Microscopy and pH measurement of stool

These assessments were performed on day 0, day 3, and day 5 for all participating patients as part of the standard protocols at the Microbiology Department of Vietnam National Children’s Hospital. These procedures encompassed: (i) Examination of fresh blood smears and sedimentation rate analysis to ascertain the presence of erythrocytes and leukocytes in stool samples. The results were categorized into different levels, including negativity (-), trace (+), mild (++), moderate (+++), and severe positivity (++++); (ii) Stool pH measurement was performed on the supernatant obtained after processing fresh stool samples. Samples were diluted (1:10) with sterile saline, homogenized, and centrifuged to produce a clear supernatant. The pH was measured immediately using a calibrated digital pH meter, with triplicate measurements averaged for accuracy; values *≤* 5.5 were considered abnormal. This criterion served as both a screening measure to ensure the selection criteria and a means of balancing participant allocation between groups. These tests were executed following ISO 15189:2012 standards and were part of the routine procedures conducted at the Department of Microbiology, Vietnam National Children’s Hospital.

### Multiplex real-time RT/PCR assay

The QIAstat-Dx Gastrointestinal Panel real-time RT/PCR (Qiagen, USA) was routinely employed on day 0 at the Department of Molecular Biology for Infectious Diseases, Vietnam National Children’s Hospital. This assay, guided by the manufacturer on a fully automated analysis system, was used for the purpose of detecting 24 intestinal pathogens in stool samples to provide guidance for appropriate treatment strategies. The panel protocol exhibited high sensitivity and specificity and was standardized according to ISO 15189:2012 criteria. The detected pathogens included: ^1^*Entamoeba histolytica*, ^2^*Cryptosporidium spp.*, ^3^*Giardia lamblia*, ^4^*Cyclospora cayetanensis*, ^5^*Vibrio vulnificus*, ^6^*V. parahaemolyticus*, ^7^*V. cholerae*, ^8^*Campylobacter spp. (C. jejuni*,* C. upsaliensis*,* C. coli)*, ^9^*Salmonella spp.*^10^, *Clostridium difficile (tcdA/tcdB)*, ^11^*Yersinia enterocolitica*, ^12^*Enterotoxigenic E. coli (ETEC)*, ^13^*Enteropathogenic E. coli (EPEC)*, ^14^*Enteroaggregative E. coli (EAEC)*, ^15^*Shiga-like toxin-producing E. coli (STEC)*, ^16^*Shiga toxin-producing E. coli (STEC) serotype O157:H7*, ^17^*Enteroinvasive E. coli (EIEC)*, ^18^*Shigella*, ^19^*Plesiomonas shigelloides*, ^20^Human Adenovirus F40/F41, ^21^Norovirus GI, Norovirus GII, ^22^Rotavirus A, ^23^Astrovirus, and ^24^Sapovirus GI/GII/ GIV/GV. The procedure involved using 25–100 mg of unpreserved stool per mL of Cary-Blair transport medium, transferring 200 µl of the sample into the QIAstat-Dx Gastrointestinal Panel Cartridge. The barcodes of the sample and the QIAstat-Dx Gastrointestinal Panel Cartridge were scanned using the QIAstat-Dx Analyzer 1.0, and the analysis was initiated. The protocol adhered to ISO 15189:2012 standards and was regularly employed in the Department of Molecular Biology for Infectious Diseases, Vietnam National Children’s Hospital.

Simultaneously, another real-time PCR SYBR Green assay was carried out on stool samples on day 0, day 5, and the discharge day (if later than day 5) to confirm the presence of *B. clausii*,* B. clausii*, and *B. coagulans*. This cross-check was conducted to ensure the proper usage of probiotics or placebo in the experimental and control groups, respectively. Specific primers used for amplification of *B. clausii*,* B. clausii*, and *B. coagulans* was presented in Table S5. The real-time PCR conditions were as follows: 95 °C for 10 min, amplification for 40 cycles at 95 °C for 15 s, 60 °C for 20 s, 72 °C for 30 s. The read-out standardization for *B. subtilis*, *B. clausii*, and *B. coagulans* analysis was set at C_t_ <35 to confirm true positives. The protocol was developed following ISO 17025:2017 guidelines and applied solely for research purposes at the Department of Molecular Biology for Infectious Diseases, Vietnam National Children’s Hospital.

### ELISA for cytokine and IgA levels

Enzyme-linked immunosorbent assays (ELISA) were performed on stool and blood samples collected on both day 0 and day 5 at the Department of Molecular Biology for Infectious Diseases at the Vietnam National Children’s Hospital. These assays aimed to determine the levels of pro-inflammatory cytokines (IL-6, IL-8, TNF-α, IL-17, and IL-23) and the anti-inflammatory cytokine IL-10 in blood samples, as well as IgA levels in stool samples. These analyses were conducted to assess changes in immune-related indicators throughout the treatment. The quantification of pro/anti-inflammatory cytokines and IgA levels was carried out using ELISA kits in accordance with the manufacturer’s instructions, specifically using kits from R&D Systems (MN, US). The measurements of the samples were performed using the SpectraMax Plus384 Microplate Reader system, and the data were subsequently analyzed using SoftMax Pro 6.3 software, developed by Molecular Devices (CA, US).

### 16S rRNA metagenomic analysis of bacterial species in stool

Stool samples were collected from patients with persistent diarrhea and healthy children for 16S rRNA metagenome analysis. These children, who visited the Pediatric Outpatient Center at the National Children’s Hospital their regular health check-ups, underwent examination and screening by doctors. A diagnosis of good health was established based onpredefined inclusion and exclusion criteria for study participation.

Inclusion criteria:

1. Age-matched (3–24 months) with study group.
2. Normal growth parameters (WHO Z-scores ± 2 SD for weight-for-age, height-for-age, and weight-for-height).
3. Normal developmental milestones.
4. Complete vaccination status per national guidelines.

Exclusion criteria included:

1. Recent medication use (antibiotics, probiotics, prebiotics, acid suppressants) within 2 months.
2. History of chronic diseases or gastrointestinal disorders.
3. Family history of inflammatory bowel disease or celiac disease.
4. Preterm birth (< 37-week gestation).
5. Maternal history of chronic diseases or obstetric complications.

All parents of healthy children who participated in the study were fully informed about the study and provided consent by signing the informed consent form. Fifteen stool samples from Healthy children (Healthy group, *n =* 15) and sixteen stool samples from each experimental group (*n* = 16) including: Control group day 0, Control group day 5, Dia30 group day 0, and Dia30 group day 5, were collected at day 0 and day 5, as well as healthy children serving as a reference group. The stool samples were randomly selected in a stratified manner considering indices such as gestational age (full-term birth), age (in months), gender (male, female), and mode of delivery (vaginal delivery, cesarean section) to obtain data reflecting the microbiota of representative participants in this study. They were used for extraction of total DNA using a DNAeasy Mini Kit (Qiagen, Stockach, Germany). The V3–V4 hypervariable region of the 16S rRNA gene was amplified using the Herculase II Fusion DNA Polymerase Nextera XT Index Kit V2, and the 300 bp paired-end DNA libraries were constructed using the 16S Metagenomic Sequencing Library Preparation kit. The 16S rRNA library sequencing was performed by Macrogene Inc. (Seoul, Republic of Korea) on a Illumina MiSeq platform (Illumina, San Diego, CA, USA), and the raw data were quality checked and base calling was conducted using Real Time Analysis (RTA). The CD-HIT-out-Miseq package was used to trim the fastq reads, filter out the short reads, and remove noise sequences^49^. Ambiguous and chimeras reads were identified and removed by rDnaTools (PacBio, USA). Remaining reads were clustered into operational taxonomic units (OTUs), using a greedy algorithm at a cut-off level of 97% sequence similarity. Qiime version 1.9.1.1 package was used for diversity analysis, including OTUs abundance and alpha rarefaction, taxonomy diversity, and beta diversity. The minimum number of reads for each group was 50,000 and QV20 quality scores exceeding 99%, ensuring robustness for subsequent sequence analysis. All bioinformatics work was performed by the technical service of Macrogene Inc. (Seoul, Republic of Korea). Further microbiota analysis and visualization were performed by an R package *microbiome*^50^.

### Statistical analysis

The tabular analysis was performed on dichotomous variables using the Chi-Square test or *t*-test. In cases when the expected value of any cell is below five, Fisher’s exact test was used. The Mann-Whitney test was applied for comparing two independent (continuous or ordinal) variables, the Wilcoxon signed-rank test for assessing two dependent groups, and the Kruskal-Wallis test for analyzing more than two independent groups in cases where the data deviated from a normal distribution. All statistical and graphical analyses were performed on GraphPad Prism v8.4.3 software (GraphPad Software, CA, USA) and R software.

## Supplementary Information

Below is the link to the electronic supplementary material.

Supplementary Material 1

## Data Availability

The study protocol and the datasets used and analysed during the current study available from the corresponding author on reasonable request. The sequence data supporting the findings of this study have been deposited in the National Center for Biotechnology Information (NCBI) database under BioProject PRJNA1231769, accessible at https://www.ncbi.nlm.nih.gov/bioproject/PRJNA1231769/.
