# Supplementary material for: High-dose multi-strain Bacillus probiotics enhance treatment and reduce antibiotic usage in children with persistent diarrhea through immune and microbiota modulation
Source: Sci Rep. 2025 Aug 18;15:30231. doi: 10.1038/s41598-025-15199-y (PMC12361499; doi:10.1038/s41598-025-15199-y)
Supplement: Supplementary file 1 — Supplementary Material 1 [file 41598_2025_15199_MOESM1_ESM.pdf]

Supplementary Data

Table S1. Treatment therapy during hospitalization

Characteristics	Control group ($N = 49$)	DIA30 group ($N = 48$)
Routine treatment	- Antibiotics: Oral (e.g., Zithromax or Ciprofloxacin) or intervention (e.g., Ceftriaxone, Ciprofloxacin, Metronidazole, or Vancomycin) drugs<br>- Oral rehydration Solution<br>- Zinc gluconate	
Supportive treatment	Reverse osmotic (RO) water	RO water plus *B. subtilis, B. clausii,* and *B. coagulans* at ≥ 5 billion CFU/5 mL (LiveSpo DIA30)
Nutritional intake	All children receive nutrition tailored to their age group:<br>• Infants under 6 months are encouraged to breastfeed. If formula milk is required, lactose-free milk or hydrolyzed formula is recommended. If there is no improvement observed symptoms, such as those related to lactose intolerance, with lactose-free milk after one week, switching to a hydrolyzed formula is advised.<br>• For children older than 6 months, breastfeeding is complemented with a balanced diet. This includes lactose-free options with reduced starch content such as cooked cereal, vegetables, oil, and glucose. Milk protein should be substituted with alternative sources like chicken, egg, or protein hydrolysate for children who cannot tolerate milk protein. Ensure that at least 10% of calorie intake is derived from protein.<br>The total estimated calorie intake is approximately 100 calories per kilogram daily.	

Table S2. Microbiological and biochemical characterization of *B. subtilis* ANA48, *B. clausii* ANA39, and *B. coagulans* ANA40

**Characteristics**	***B. subtilis* ANA48**	***B. clausii* ANA39**	***B. coagulans* ANA40**
Sporulation efficiency (%)	98	90	90
Heat stability of spores (°C)	80	65	60
Width size of vegetative cell (μm)	< 1 μm	< 1 μm	< 1 μm
Amylase	+++	+++	++++
Caseinase	++++	++++	-
Lipase	+	++	-
Catalase	++	+	+++
Gelatinase	++	+	++
Optimal temperature (°C)	37	35	44
Optimal pH	7.5	8.0	6.5
6.5% NaCl, 50°C	+	-	-
Aerobic/Anaerobic	++++	+++	+++
Anerobic	++++	+++	+++
Hemolysis	γ (No)	γ (No)	γ (No)
VP Test	+	-	+
Closest match*	*B. subtilis* (100%)	*B. clausii* (99.5%)	*B. coagulans* (99.4%)

-, negative; +, weak or positive; ++, average; +++, good/high; ++++, very good/very high.

**Using 16S rDNA sequence analysis in this work. The similarity score is shown in brackets.*

Table S3. Antibiotic susceptibility of *B. subtilis* ANA48, *B. clausii* ANA39, and *B. coagulans* ANA40

**Antibiotic discs (µg)***	***B. subtilis* ANA48**	***B. clausii* ANA39**	***B. coagulans* ANA40**
Ampicillin (10)	28.42 ± 0.46 (S)	27.04 ± 1.3 (S)	28.30 ± 0.21 (S)
Chloramphenicol (30)	31.25 ± 0.81 (S)	18.47 ± 0.20 (S)	30.11 ± 0.39 (S)
Ciprofloxacin (5)	30.96 ± 0.41 (S)	30.89 ± 0.61 (S)	25.10 ± 0.31 (S)
Clindamycin (2)	17.12 ± 0.08 (S)	0 (R)	25.12 ± 1.08 (S)
Cotrimoxazole (25)	32.43 ± 0.03 (S)	34.50 ± 0.95 (S)	25.71 ± 0.50 (S)
Erythromycin (15)	30.06 ± 0.13 (S)	0 (R)	22.60 ± 0.51 (S)
Gentamicin (10)	29.50 ± 0.16 (S)	28.22 ± 0.39 (S)	24.41 ± 0.31 (S)
Kanamycin (30)	29.32 ± 0.31 (S)	24.84 ± 0.04 (S)	26.4 ± 0.03 (S)
Neomycin (30)	24.17 ± 0.14 (S)	24.91 ± 0.13 (S)	21.40 ± 0.30 (S)
Rifampicin (30)	22.51 ± 0.09 (S)	39.06 ± 0.68 (S)	39.87 ± 0.31 (S)
Streptomycin (10)	14.03 ± 0.82 (I)	6.51 ± 0.46 (R)	16.20 ± 0.40 (S)
Tetracycline (30)	26.05 ± 0.26 (S)	27.99 ± 0.14 (S)	35.50 ± 1.10 (S)
Trimethoprim (5)	32.50 ± 0.14 (S)	39.83 ± 0.72 (S)	22.81 ± 0.40 (S)
Vancomycin (30)	16.55 ± 0.45 (S)	22.41 ± 0.22 (S)	20.41 ± 0.22 (S)
Azithromycin (15)	26.32 ± 0.11 (S)	0 (R)	22.07 ± 0.48 (S)
Clarithromycin (15)	31.16 ± 0.07 (S)	0 (R)	24.49 ± 0.53 (S)

**Antibiotic-impregnated discs (6 mm) with amount in µg shown in brackets.*

+Diameter of inhibition zones from three individual experiments. S, sensitive; I, intermediate resistant; R, resistant.

Table S4. Sequence analysis of toxin genes in *B. subtilis* ANA48, *B. clausii* ANA39, and *B. coagulans* ANA40 genome

No	Gene	Specific sequence amplified by PCR			Number of gene detected by whole genome sequencing		
		***B. subtilis* ANA48**	***B. clausii* ANA39**	***B. coagulans* ANA40**	***B. subtilis* ANA48**	***B. clausii* ANA39**	***B. coagulans* ANA40**
1	Hemolysin B (*hbl*B)	ND	ND	ND	0	0	0
2	Non-hemolytic enterotoxin A (*nhe*A)	ND	ND	ND	0	0	0
3	*nhe*B	ND	ND	ND	0	0	0
4	*nhe*C	ND	ND	ND	0	0	0
5	Cytotoxin K (*cyt*K)	ND	ND	ND	0	0	0

Table S5. Specific primers for real time PCR SYBR Green amplification of *B. subtilis*, *B. clausii*, and *B. coagulans*

Species	Primer name	Sequence (5’-3’)	Length (bp)	Reference
B. subtilis	Subtilis-F	ACCATTGCGGTAGGTGCG	18	Sadeghi *et al.,* 2014
	Subtilis-R	GCGTTTGTCCAAGTCGGG	18	
B. clausii	Clausii-F	AATTTTTACCGCCCTCAAG	19	Perotti *et al.,* 2006
	Clausii-R	ACTTTTGGAACATGCCGAAC	20	
B. coagulans	Coagulans-F	AGTGCCGTTCGAACAGGGCGGCGCC	18	Majeed *et al.*, 2017
	Coagulans-R	AGCCGCCTGCGCGCGCTTTACGCCC	22	

References

1. Sadeghi A, Ali Mortazavi S, Reza Bahrami A, et al (2014), "Designing a SYBR Green Absolute Real time PCR Assay for Specific Detection and Quantification of *Bacillus subtilis* in Dough Used for Bread Making". J Cell Mol Res 6:83–92.
2. Perotti M, Mancini N, Cavallero A, et al (2006) "Quantitation of *Bacillus clausii* in biological samles by real-times polymerase chain reaction". J Microbiol Method 65:632-636.
3. Majeed M, Nagabhushanam K, Mundkur L, Paulose S, Divakar H, Rao S, Arumugam S. Probiotic modulation of gut microbiota by *Bacillus coagulans* MTCC 5856 in healthy subjects: A randomized, double-blind, placebo-control study. Medicine 2023;102:20(e33751).

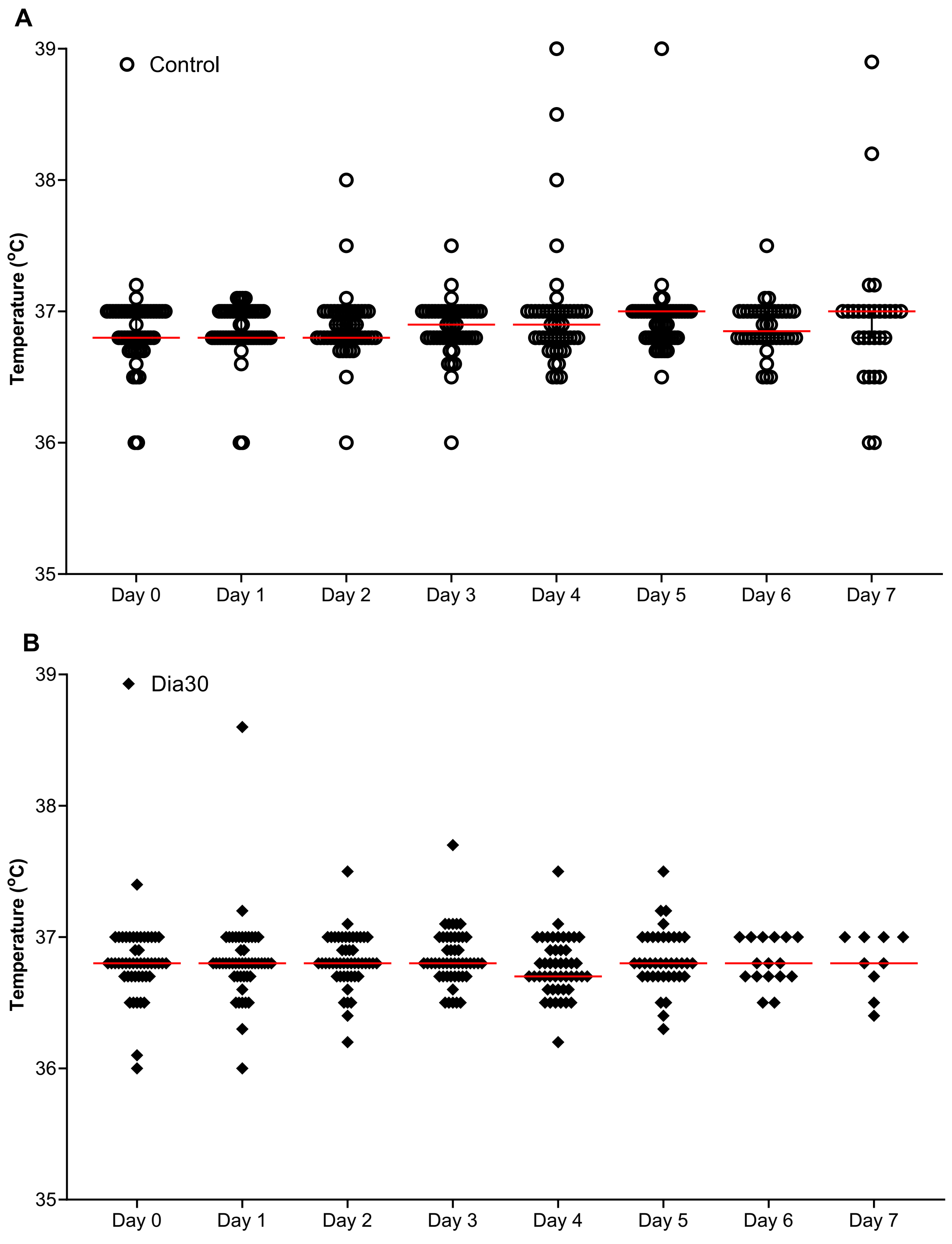

Fig. S1. Average values of recorded changes in temperature between before and after oral administration with RO water (A) and LiveSpo DIA30 at the daily dosages of 20-30 billion CFU (B), over 7 days of measurement.

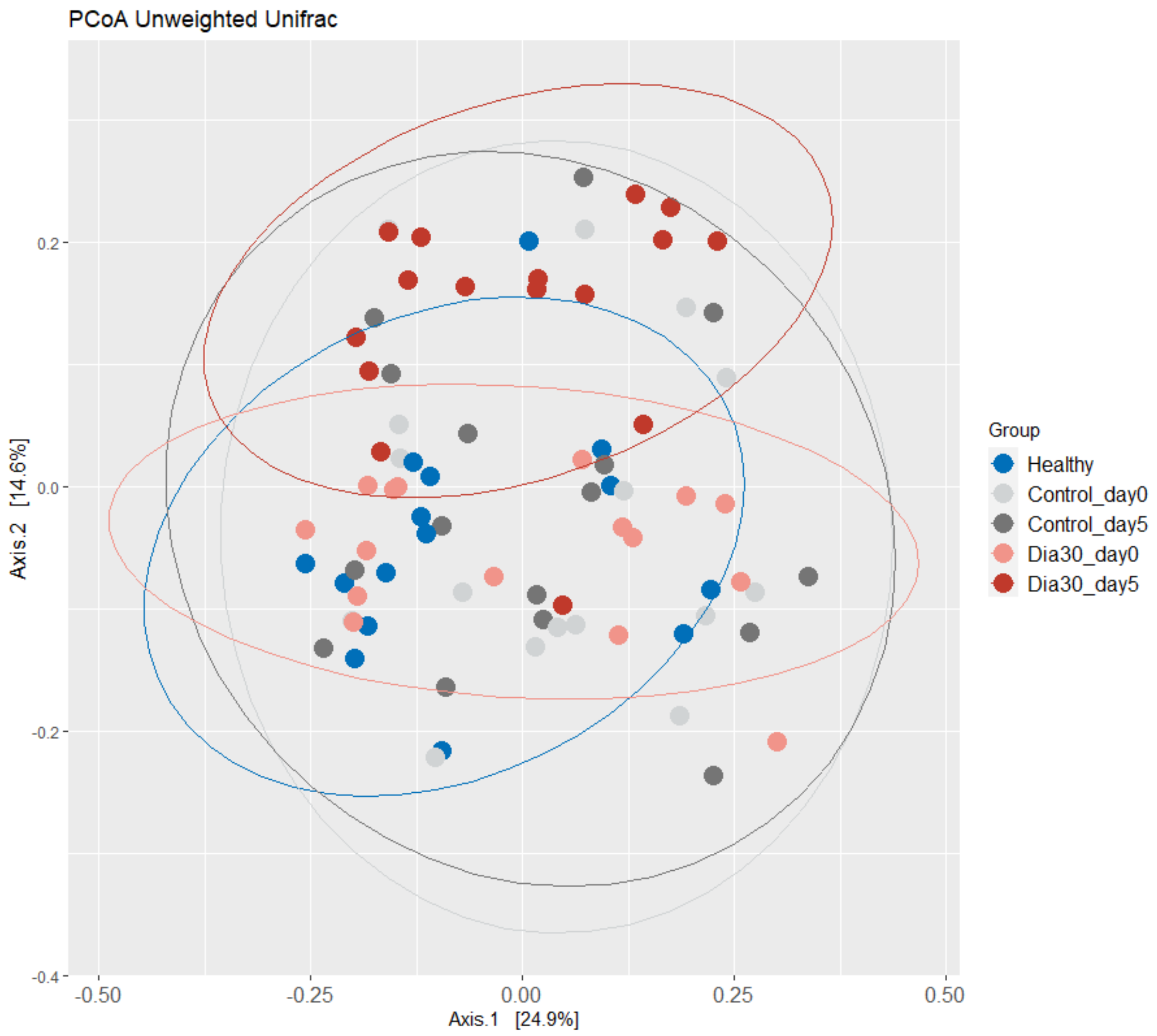

Fig. S2. PCoA analysis of beta diversity of 16S rRNA microbiota of stool samples between healthy children group and Control, Dia30 groups at day 5 compared to day 0.

A.

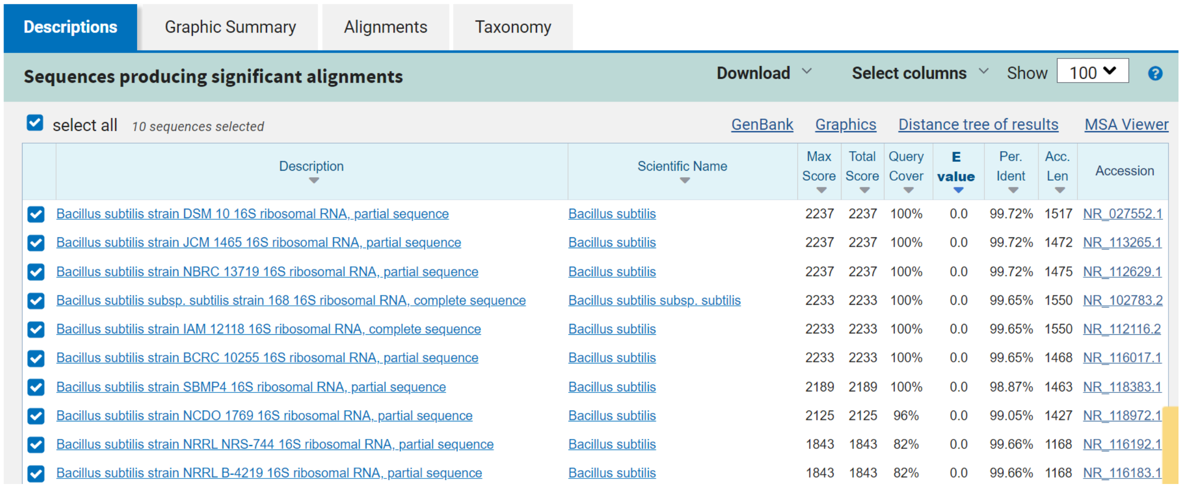

Descriptions | Graphic Summary | Alignments | Taxonomy

Sequences producing significant alignments — Download — Select columns — Show 100

select all 10 sequences selected — GenBank Graphics Distance tree of results MSA Viewer

	Description	Scientific Name	Max Score	Total Score	Query Cover	E value	Per. Ident	Acc. Len	Accession
☑	Bacillus subtilis strain DSM 10 16S ribosomal RNA, partial sequence	Bacillus subtilis	2237	2237	100%	0.0	99.72%	1517	NR_027552.1
☑	Bacillus subtilis strain JCM 1465 16S ribosomal RNA, partial sequence	Bacillus subtilis	2237	2237	100%	0.0	99.72%	1472	NR_113265.1
☑	Bacillus subtilis strain NBRC 13719 16S ribosomal RNA, partial sequence	Bacillus subtilis	2237	2237	100%	0.0	99.72%	1475	NR_112629.1
☑	Bacillus subtilis subsp. subtilis strain 168 16S ribosomal RNA, complete sequence	Bacillus subtilis subsp. subtilis	2233	2233	100%	0.0	99.65%	1550	NR_102783.2
☑	Bacillus subtilis strain IAM 12118 16S ribosomal RNA, complete sequence	Bacillus subtilis	2233	2233	100%	0.0	99.65%	1550	NR_112116.2
☑	Bacillus subtilis strain BCRC 10255 16S ribosomal RNA, partial sequence	Bacillus subtilis	2233	2233	100%	0.0	99.65%	1468	NR_116017.1
☑	Bacillus subtilis strain SBMP4 16S ribosomal RNA, partial sequence	Bacillus subtilis	2189	2189	100%	0.0	98.87%	1463	NR_118383.1
☑	Bacillus subtilis strain NCDO 1769 16S ribosomal RNA, partial sequence	Bacillus subtilis	2125	2125	96%	0.0	99.05%	1427	NR_118972.1
☑	Bacillus subtilis strain NRRL NRS-744 16S ribosomal RNA, partial sequence	Bacillus subtilis	1843	1843	82%	0.0	99.66%	1168	NR_116192.1
☑	Bacillus subtilis strain NRRL B-4219 16S ribosomal RNA, partial sequence	Bacillus subtilis	1843	1843	82%	0.0	99.66%	1168	NR_116183.1

B.

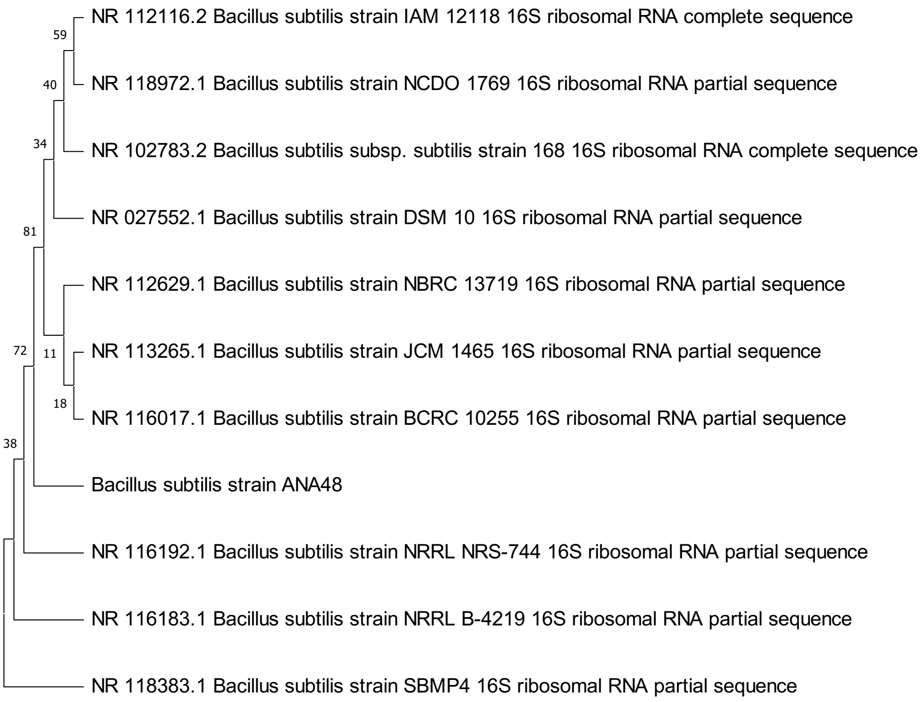

Fig. S3. BLAST analysis (A) and Phylogenetic tree (B) of *B. subtilis* ANA48 (accession no. PP851085 in NCBI)

A.

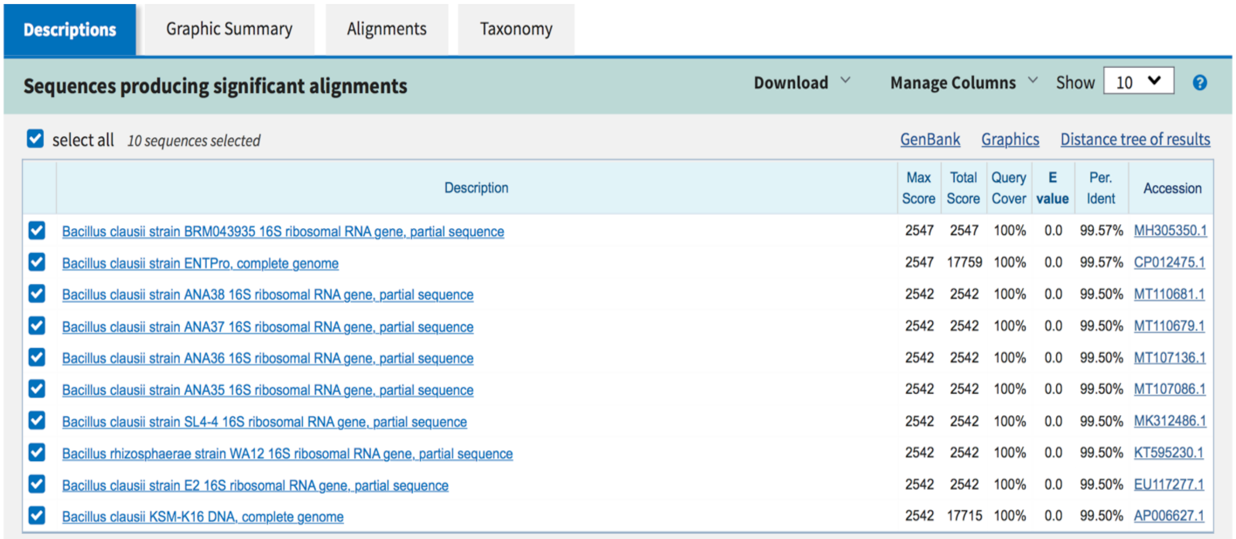

Descriptions | Graphic Summary | Alignments | Taxonomy

Sequences producing significant alignments Download Manage Columns Show 10

select all *10 sequences selected* GenBank Graphics Distance tree of results

	Description	Max Score	Total Score	Query Cover	E value	Per. Ident	Accession
☑	Bacillus clausii strain BRM043935 16S ribosomal RNA gene, partial sequence	2547	2547	100%	0.0	99.57%	MH305350.1
☑	Bacillus clausii strain ENTPro, complete genome	2547	17759	100%	0.0	99.57%	CP012475.1
☑	Bacillus clausii strain ANA38 16S ribosomal RNA gene, partial sequence	2542	2542	100%	0.0	99.50%	MT110681.1
☑	Bacillus clausii strain ANA37 16S ribosomal RNA gene, partial sequence	2542	2542	100%	0.0	99.50%	MT110679.1
☑	Bacillus clausii strain ANA36 16S ribosomal RNA gene, partial sequence	2542	2542	100%	0.0	99.50%	MT107136.1
☑	Bacillus clausii strain ANA35 16S ribosomal RNA gene, partial sequence	2542	2542	100%	0.0	99.50%	MT107086.1
☑	Bacillus clausii strain SL4-4 16S ribosomal RNA gene, partial sequence	2542	2542	100%	0.0	99.50%	MK312486.1
☑	Bacillus rhizosphaerae strain WA12 16S ribosomal RNA gene, partial sequence	2542	2542	100%	0.0	99.50%	KT595230.1
☑	Bacillus clausii strain E2 16S ribosomal RNA gene, partial sequence	2542	2542	100%	0.0	99.50%	EU117277.1
☑	Bacillus clausii KSM-K16 DNA, complete genome	2542	17715	100%	0.0	99.50%	AP006627.1

B.

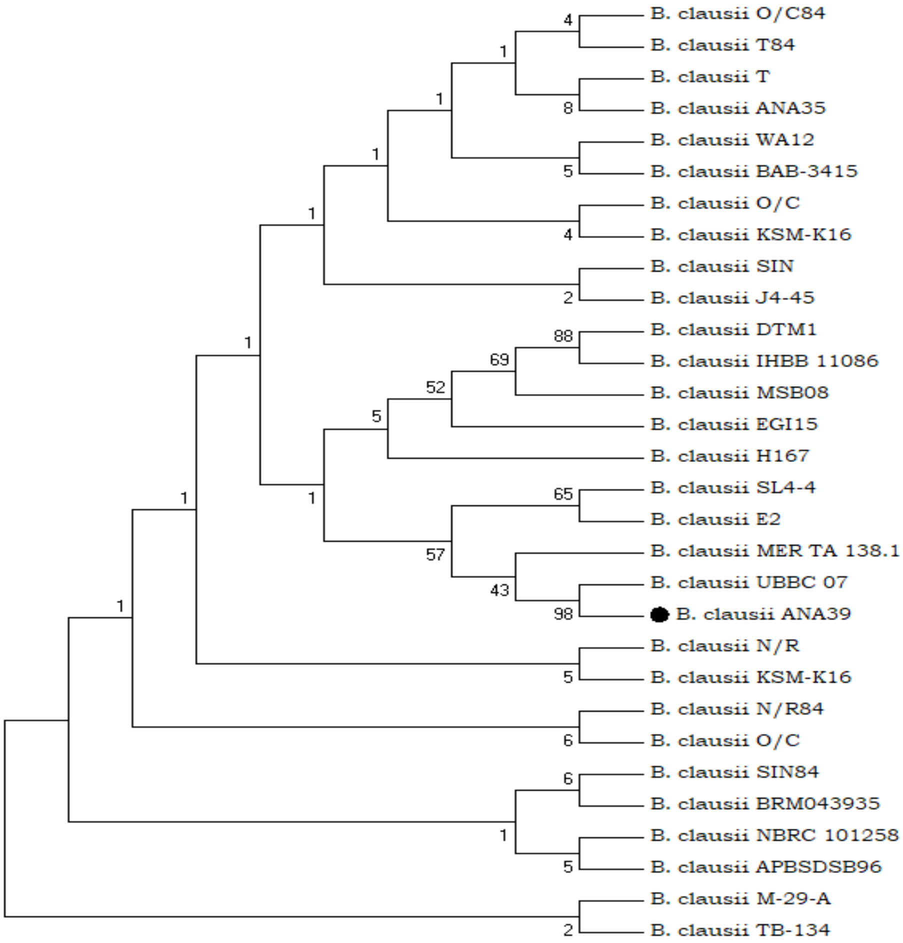

Fig. S4. BLAST analysis (A) and Phylogenetic tree (B) of *B. clausii* ANA39 (accession no. MT275656.1 in NCBI)

A.

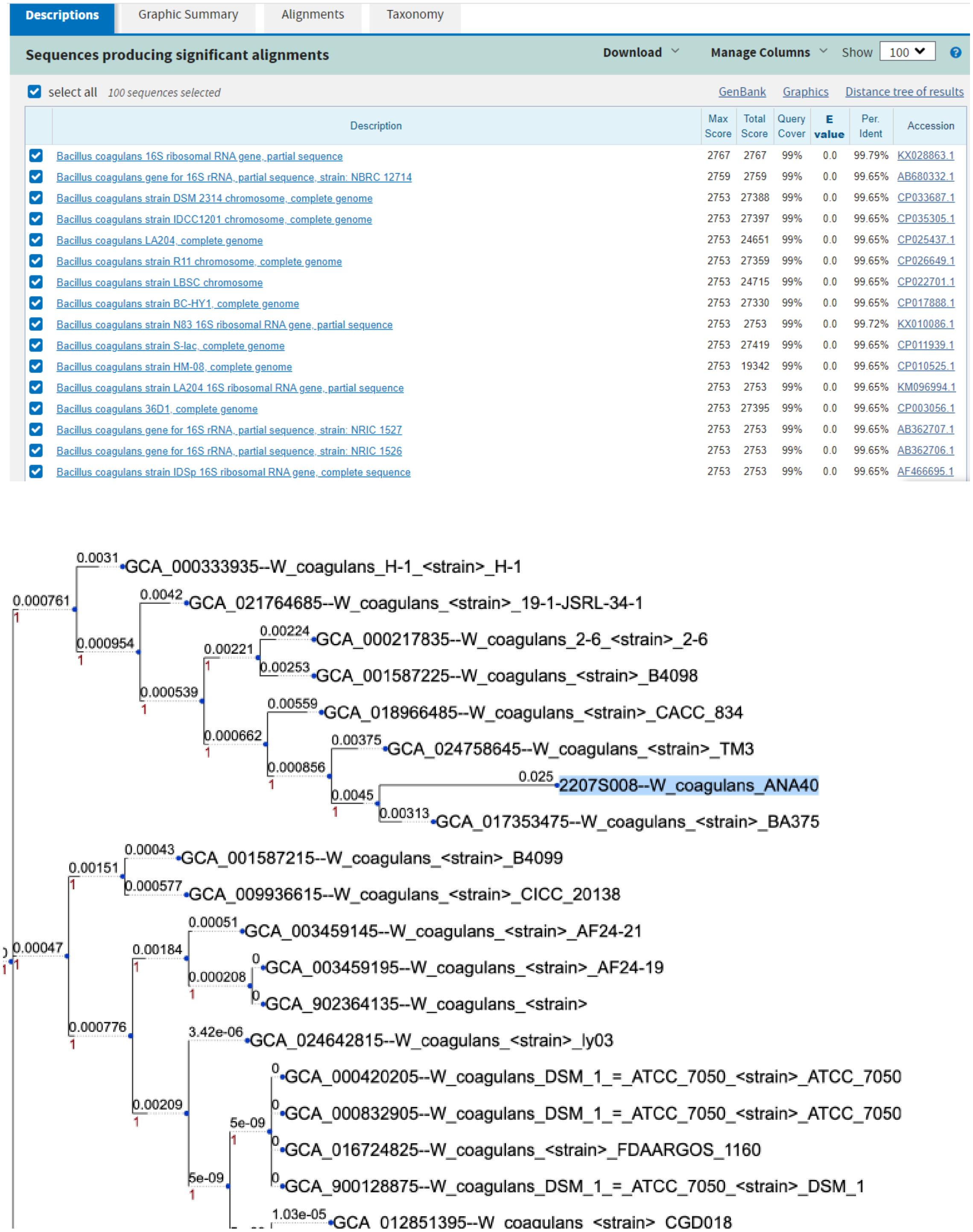

Descriptions | Graphic Summary | Alignments | Taxonomy

Sequences producing significant alignments — Download — Manage Columns — Show 100

select all 100 sequences selected — GenBank Graphics Distance tree of results

	Description	Max Score	Total Score	Query Cover	E value	Per. Ident	Accession
☑	Bacillus coagulans 16S ribosomal RNA gene, partial sequence	2767	2767	99%	0.0	99.79%	KX028863.1
☑	Bacillus coagulans gene for 16S rRNA, partial sequence, strain: NBRC 12714	2759	2759	99%	0.0	99.65%	AB680332.1
☑	Bacillus coagulans strain DSM 2314 chromosome, complete genome	2753	27388	99%	0.0	99.65%	CP033687.1
☑	Bacillus coagulans strain IDCC1201 chromosome, complete genome	2753	27397	99%	0.0	99.65%	CP035305.1
☑	Bacillus coagulans LA204, complete genome	2753	24651	99%	0.0	99.65%	CP025437.1
☑	Bacillus coagulans strain R11 chromosome, complete genome	2753	27359	99%	0.0	99.65%	CP026649.1
☑	Bacillus coagulans strain LBSC chromosome	2753	24715	99%	0.0	99.65%	CP022701.1
☑	Bacillus coagulans strain BC-HY1, complete genome	2753	27330	99%	0.0	99.65%	CP017888.1
☑	Bacillus coagulans strain N83 16S ribosomal RNA gene, partial sequence	2753	2753	99%	0.0	99.72%	KX010086.1
☑	Bacillus coagulans strain S-lac, complete genome	2753	27419	99%	0.0	99.65%	CP011939.1
☑	Bacillus coagulans strain HM-08, complete genome	2753	19342	99%	0.0	99.65%	CP010525.1
☑	Bacillus coagulans strain LA204 16S ribosomal RNA gene, partial sequence	2753	2753	99%	0.0	99.65%	KM096994.1
☑	Bacillus coagulans 36D1, complete genome	2753	27395	99%	0.0	99.65%	CP003056.1
☑	Bacillus coagulans gene for 16S rRNA, partial sequence, strain: NRIC 1527	2753	2753	99%	0.0	99.65%	AB362707.1
☑	Bacillus coagulans gene for 16S rRNA, partial sequence, strain: NRIC 1526	2753	2753	99%	0.0	99.65%	AB362706.1
☑	Bacillus coagulans strain IDSp 16S ribosomal RNA gene, complete sequence	2753	2753	99%	0.0	99.65%	AF466695.1

B.

Fig. S5. BLAST analysis (A) and Phylogenetic tree (B) of *B. coagulans* ANA40 (accession no. MT734108 in NCBI)
